# Supplementary material for: ERK1/2 Activity Is Critical for the Outcome of Ischemic Stroke
Source: Int J Mol Sci. 2022 Jan 9;23(2):706. doi: 10.3390/ijms23020706 (PMC8776221; doi:10.3390/ijms23020706)

Supplementary Information accompanying:

ERK1/2 activity is critical for the outcome of ischemic stroke

Constanze Schanbacher[1,2]*, Michael Bieber[3]*, Yvonne Reinders[2], Deya Cherpokova[4,5], Christina Teichert[2], Bernhard Nieswandt[4,5], Albert Sickmann[2], Christoph Kleinschnitz[6], Friederike Langhauser[6]#, Kristina Lorenz[1,2]#

[1]Institute of Pharmacology and Toxicology, University of Würzburg, Würzburg, Germany

[2]Leibniz-Institut für Analytische Wissenschaften - ISAS - e.V., Dortmund, Germany

[3]Department of Neurology, University Hospital Würzburg, Würzburg, Germany

[4]Institute of Experimental Biomedicine I, University Hospital Würzburg, Würzburg, Germany

[5]Rudolf Virchow Center, University of Würzburg, Würzburg, Germany

[6]Department of Neurology and Center for Translational Neuro- and Behavioral Sciences (C-TNBS), University Hospital Essen, Essen, Germany

* Authors contributed equally

# Corresponding authors

Correspondence to

lorenz@toxi.uni-wuerzburg.de or friederike.langhauser@uk-essen.de

Supplementary Information

Supplementary Figures

Figure S1

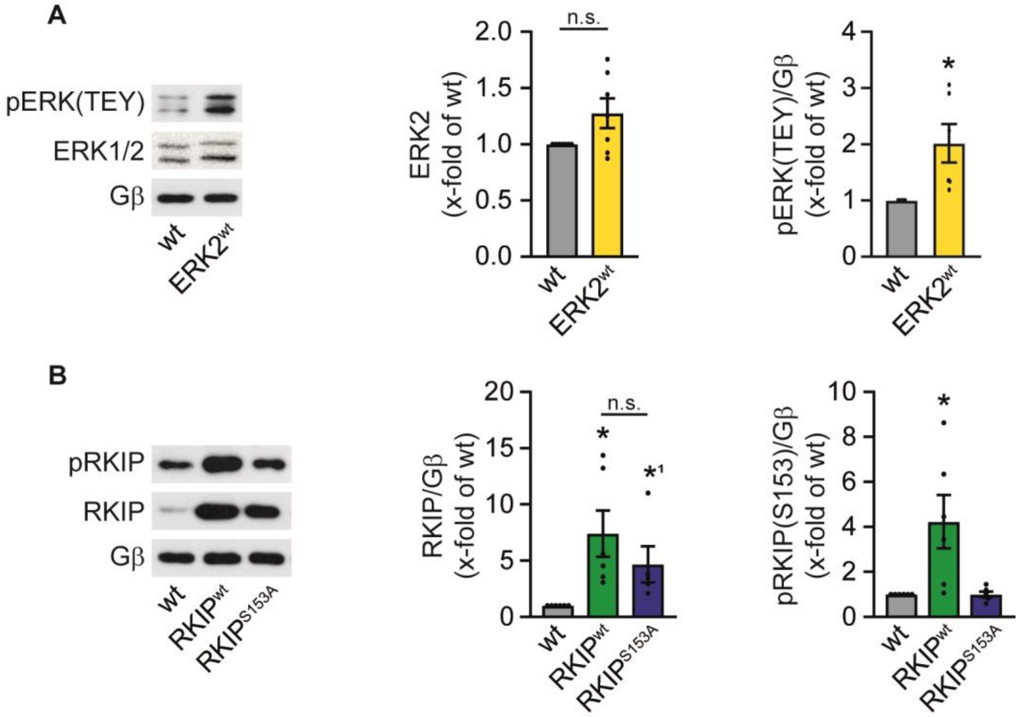

Figure S1: Protein expression and phosphorylation levels of ERK1/2 and RKIP in the brain of wt, ERK2^{wt}, RKIP^{wt} and RKIP^{S153A} mice. Immunoblot analysis of ERK1/2 (n = 7) and pERK(TEY) (n = 6) (**A**) as well as RKIP (wt, RKIP^{wt}: n = 6; RKIP^{S153A}: n = 5) and pRKIP (n = 6) (**B**) in brain tissue of the contralateral basal ganglia of wild-type (wt) mice, mice with ubiquitous overexpression of ERK2^{wt} (ERK2^{wt}), RKIP^{wt} (RKIP^{wt}) or a phosphorylation-deficient mutant of RKIP^{S153A} (RKIP^{S153A}) 24 h after transient middle cerebral artery occlusion. Shown are representative western blots and quantitative analysis. Gβ was used as loading control. Error bars are mean ± SEM; n represents the number of animals. *$p < 0.05$ versus wt in an ANOVA test. *$^1p < 0.05$ versus wt in a t-test. n.s. not significant.

Figure S2

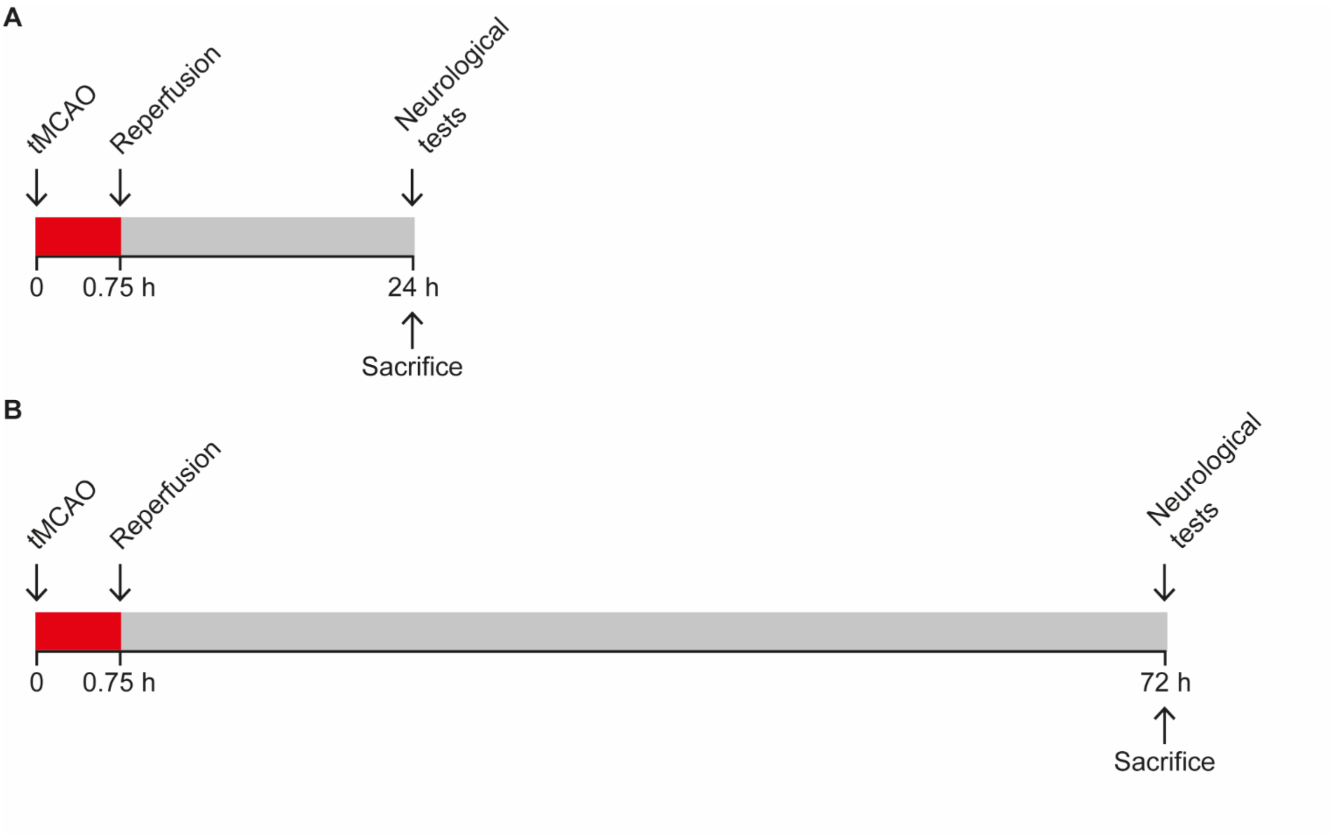

Figure S2: Experimental design. Male FVB/N mice with ubiquitous overexpression of ERK2^{wt}, RKIP^{wt} or a phosphorylation-deficient mutant of RKIP^{S153A} and their gender matched wild-type littermates were exposed to 45 minutes of transient middle cerebral artery occlusion (tMCAO) to induce focal cerebral ischemia followed by reperfusion. 24 h (**A**) or 72 h (**B**) after tMCAO neurological deficits were evaluated by Bederson score and grip test and animals were sacrificed. Brain samples were taken and used for further analyses.

Supplementary Tables

Table S1

Accession Number	Protein Name	ratio wt bgc vs. wt bgi	p value
Q9ERT9	Protein phosphatase 1 regulatory subunit 1A	5.20412509	0.00064634
Q8CC35	Synaptopodin	4.77400451	0.03952532
Q3V0I2	Proline-rich protein 7	3.98336744	0.04475555
Q3UHD1	Adhesion G protein-coupled receptor B1	3.85947823	0.00052681
Q9D1J3	SAP domain-containing ribonucleoprotein	3.67534009	0.0027902
Q9CY57	Chromatin target of PRMT1 protein	3.58097426	2.3839E-05
P41242	Megakaryocyte-associated tyrosine-protein kinase	3.41666886	0.00999621
Q810A7	ATP-dependent RNA helicase DDX42	3.37183961	0.02309052
Q9Z275	Retinaldehyde-binding protein 1	3.33666989	0.01987248
Q9CQ80	Vacuolar protein-sorting-associated protein 25	3.1056536	0.00026643
Q6PGL7	WASH complex subunit 2	3.03184825	0.00117529
O89086	RNA-binding protein 3	2.9828436	0.01618581
Q923D2	Flavin reductase (NADPH)	2.95082754	0.04485723
Q80WQ2	Protein VAC14 homolog	2.88170361	0.04824103
P20357	Microtubule-associated protein 2	2.87638513	0.03999333
Q9R0N3	Synaptotagmin-11	2.86963247	0.00911254
Q8CCJ4	APC membrane recruitment protein 2	2.79083172	0.00095877
P0C7L0	WAS/WASL-interacting protein family member 3	2.58635915	0.02572165
Q8C2Q3	RNA-binding protein 14	2.58500557	0.01336703
Q68ED7	CREB-regulated transcription coactivator 1	2.55389925	0.00463423
Q80X50	Ubiquitin-associated protein 2-like	2.5159157	0.00661382
Q62448	Eukaryotic translation initiation factor 4 gamma 2	2.4593551	0.00708659
Q8C3Q5	Protein shisa-7	2.44610682	0.00508189
Q9CZN4	Protein shisa-9	2.42060454	0.02055149
Q80TE7	Leucine-rich repeat-containing protein 7	2.35319575	0.00747443
Q9JM52	Misshapen-like kinase 1	2.33680593	0.00254945
Q9CZG9	PDZ domain-containing protein 11	2.33631767	0.00075802
A2BDX3	Adenylyltransferase and sulfurtransferase MOCS3	2.28356998	0.00495197
P83510	Traf2 and NCK-interacting protein kinase	2.20311319	0.0019425
Q9DC07	LIM zinc-binding domain-containing Nebulette	2.19009665	0.00729629
A2ALU4	Protein Shroom2	2.1649366	0.01700068
P97825	Jupiter microtubule associated homolog 1	2.13203081	0.03911401
P59644	Phosphatidylinositol 4,5-bisphosphate 5-phosphatase A	2.11780563	0.02897564
Q80TK0	AP2-interacting clathrin-endocytosis protein	2.11711937	0.02490817
Q9WV69	Dematin	2.0891437	0.00285244

Accession Number	Protein Name	ratio wt bgc vs. wt bgi	p value
Q8K1R3	Polyribonucleotide nucleotidyltransferase 1, mitochondrial	2.08295674	0.04748834
Q8C0T5	Signal-induced proliferation-associated 1-like protein 1	2.06641427	0.00179711
Q58A65	C-Jun-amino-terminal kinase-interacting protein 4	2.04748599	0.01022775
Q01097	Glutamate receptor ionotropic, NMDA 2B	2.0443333	0.03178183
P62309	Small nuclear ribonucleoprotein G	2.03921902	0.04159384
Q60598	Src substrate cortactin	2.03466285	0.04322095
Q80WC7	Arf-GAP domain and FG repeat-containing protein 2	2.01840281	0.01857531
Q811P8	Rho GTPase-activating protein 32	1.9921492	0.0464889
Q9JHL1	Na(+)/H(+) exchange regulatory cofactor NHE-RF2	1.94816936	0.00345993
Q3UEB3	Poly(U)-binding-splicing factor PUF60	1.9473762	0.00489533
Q8BJF9	Charged multivesicular body protein 2b	1.93358333	0.01796072
Q8CAK3	Shiftless antiviral inhibitor of ribosomal frameshifting protein homolog	1.92799313	0.04900832
P60840	Alpha-endosulfine	1.91474236	0.00724567
Q71M36	Chondroitin sulfate proteoglycan 5	1.90727951	0.04534506
Q8R3Z5	Voltage-dependent L-type calcium channel subunit beta-1	1.90321186	0.02989799
Q924C5	Alpha-protein kinase 3	1.89268407	0.02048537
P26040	Ezrin	1.89122736	0.00083063
Q7TQH0	Ataxin-2-like protein	1.88643453	0.0050316
Q64213	Splicing factor 1	1.88033077	0.00313093
Q8K3W0	BRISC and BRCA1-A complex member 2	1.85259413	0.01119482
P34152	Focal adhesion kinase 1	1.84342814	0.00323227
Q9CQH7	Transcription factor BTF3 homolog 4	1.81767872	0.01577126
Q4U4S6	Xin actin-binding repeat-containing protein 2	1.79748372	0.02764688
Q60936	Atypical kinase COQ8A, mitochondrial	1.74152458	0.02095725
Q8BL65	Actin-binding LIM protein 2	1.73975155	0.03669039
Q922J3	CAP-Gly domain-containing linker protein 1	1.70064916	0.02790328
Q9CQV1	Mitochondrial import inner membrane translocase subunit TIM16	1.69537547	0.04138398
Q62417	Sorbin and SH3 domain-containing protein 1	1.68381234	0.00962248
Q64378	Peptidyl-prolyl cis-trans isomerase FKBP5	1.6781826	0.04664082
Q8K019	Bcl-2-associated transcription factor 1	1.67777954	0.01006402
P63166	Small ubiquitin-related modifier 1	1.65541769	0.0243469
O35927	Catenin delta-2	1.65124798	0.0331326
O70310	Glycylpeptide N-tetradecanoyltransferase 1	1.64910193	0.02433471
Q6A0A9	Constitutive coactivator of PPAR-gamma-like protein 1	1.64505086	0.03692227
O88737	Protein bassoon	1.62394475	0.01202164
Q91ZM2	SH2B adapter protein 1	1.62278435	0.01431594
Q9DBR7	Protein phosphatase 1 regulatory subunit 12A	1.61587667	0.04428091

Accession Number	Protein Name	ratio wt bgc vs. wt bgi	p value
O89023	Tripeptidyl-peptidase 1	1.59430999	0.04309287
Q6PD28	Serine/threonine-protein phosphatase 2A 56 kDa regulatory subunit beta isoform	1.55774993	0.03851172
Q99K28	ADP-ribosylation factor GTPase-activating protein 2	1.5396835	0.02210375
P70408	Cadherin-10	1.53831665	0.01223212
O08919	Numb-like protein	1.53673102	0.03197404
Q8CHU3	Epsin-2	1.52520078	0.00718283
Q6R891	Neurabin-2	1.52164122	0.02261814
Q5RJI5	Serine/threonine-protein kinase BRSK1	1.52094429	0.0011169
Q9QYR6	Microtubule-associated protein 1A	1.51640733	0.02477992
Q6PCP5	Mitochondrial fission factor	1.51426751	0.04532473
P58802	TBC1 domain family member 10A	1.50659196	0.00751549
O35226	26S proteasome non-ATPase regulatory subunit 4	1.50362662	0.01997354
P70697	Uroporphyrinogen decarboxylase	1.50259989	0.03581633
Q9D8W7	OCIA domain-containing protein 2	1.49929241	0.03485246
Q9Z172	Small ubiquitin-related modifier 3	1.49500722	0.04231473
P97797	Tyrosine-protein phosphatase non-receptor type substrate 1	1.49094958	0.01123553
P81270	Transcriptional regulator ERG	1.49093639	0.04450761
Q3UHD9	Arf-GAP with GTPase, ANK repeat and PH domain-containing protein 2	1.48905786	0.0109838
Q922V4	Pleiotropic regulator 1	1.48864341	0.03068827
Q9CY58	Plasminogen activator inhibitor 1 RNA-binding protein	1.48737772	0.00787602
Q8R5H6	Wiskott-Aldrich syndrome protein family member 1	1.47217396	0.01968891
O08579	Emerin	1.46848457	0.00112861
Q8VCE6	5'(3')-deoxyribonucleotidase, mitochondrial	1.45812516	0.0412454
Q14BB9	MAP6 domain-containing protein 1	1.45804914	0.03895054
Q8R1N4	NudC domain-containing protein 3	1.44905434	0.02031647
P10711	Transcription elongation factor A protein 1	1.44531855	0.0339959
Q8CFI0	E3 ubiquitin-protein ligase NEDD4-like	1.44361701	0.03667982
Q6P4T2	U5 small nuclear ribonucleoprotein 200 kDa helicase	1.43938975	0.03095675
Q8CDG3	Deubiquitinating protein VCIP135	1.43632284	0.00797666
Q9CTY5	Calcium uptake protein 3, mitochondrial	1.41630429	0.04104422
Q9WUK2	Eukaryotic translation initiation factor 4H	1.41365062	0.03577911
Q6PDL0	Cytoplasmic dynein 1 light intermediate chain 2	1.40397759	0.0235399
Q8VHI6	Wiskott-Aldrich syndrome protein family member 3	1.39054852	0.02948804
P10605	Cathepsin B	1.39051494	0.03958936
Q9QWY8	Arf-GAP with SH3 domain, ANK repeat and PH domain-containing protein 1	1.38958317	0.02038611
Q9JKY5	Huntingtin-interacting protein 1-related protein	1.36867425	0.01785722

Accession Number	Protein Name	ratio wt bgc vs. wt bgi	p value
Q60738	Zinc transporter 1	1.36321141	0.03218666
O70161	Phosphatidylinositol 4-phosphate 5-kinase type-1 gamma	1.35578837	0.0101605
Q99N95	39S ribosomal protein L3, mitochondrial	1.35257617	0.03130405
O88569	Heterogeneous nuclear ribonucleoproteins A2/B1	1.35012591	0.00599544
Q91XM9	Disks large homolog 2	1.34688464	0.01888034
Q6NZB0	DnaJ homolog subfamily C member 8	1.33989715	0.04477211
P62774	Myotrophin	1.33896477	0.03004042
Q9QYC0	Alpha-adducin	1.3320279	0.009899
Q9Z0P4	Paralemmin-1	1.32760679	0.03665203
P61222	ATP-binding cassette sub-family E member 1	1.31855466	0.0140073
Q8BUV3	Gephyrin	1.31096984	0.01341845
Q61771	Kinesin-like protein KIF3B	1.31003952	0.01102867
Q9CZY3	Ubiquitin-conjugating enzyme E2 variant 1	1.30094446	0.01069872
P51859	Hepatoma-derived growth factor	1.29846016	0.04422239
Q9JII5	DAZ-associated protein 1	1.29492036	0.01743697
Q9Z0P5	Twinfilin-2	1.28093247	0.01378947
Q9QYX7	Protein piccolo	1.28075723	0.03387041
Q8K0G5	EARP and GARP complex-interacting protein 1	1.28010192	0.00876746
Q9QYS9	Protein quaking	1.26313161	0.040814
Q9QYB8	Beta-adducin	1.26191203	0.00286116
P53702	Cytochrome c-type heme lyase	1.26078232	0.03797435
Q8VD37	SH3-containing GRB2-like protein 3-interacting protein 1	1.24198332	0.02294585
O70493	Sorting nexin-12	1.21470154	0.01390905
Q9JI46	Diphosphoinositol polyphosphate phosphohydrolase 1	1.20374897	0.01959568
Q9D0E1	Heterogeneous nuclear ribonucleoprotein M	1.18594404	0.01190737
Q9R0H0	Peroxisomal acyl-coenzyme A oxidase 1	1.1819556	0.01543145
Q8R326	Paraspeckle component 1	1.17911193	0.0462514
O35621	Phosphomannomutase 1	1.17714662	0.02036553
Q60631	Growth factor receptor-bound protein 2	1.17651157	0.03922862
P70296	Phosphatidylethanolamine-binding protein 1	1.16929796	0.04610836
Q8BGR6	ADP-ribosylation factor-like protein 15	1.13351573	0.04139894
P17742	Peptidyl-prolyl cis-trans isomerase A	1.1182613	0.04252818
Q5HZI9	Solute carrier family 25 member 51	1.07237345	0.03853606
Q9Z1S5	Neuronal-specific septin-3	0.90990172	0.04824856
P63005	Platelet-activating factor acetylhydrolase IB subunit alpha	0.89781391	0.03918356
P42932	T-complex protein 1 subunit theta	0.88969147	0.03171953
Q9CQH3	NADH dehydrogenase [ubiquinone] 1 beta subcomplex subunit 5, mitochondrial	0.88579696	0.01074921
P42669	Transcriptional activator protein Pur-alpha	0.88151393	0.01477786

Accession Number	Protein Name	ratio wt bgc vs. wt bgi	p value
Q9D6J6	NADH dehydrogenase [ubiquinone] flavoprotein 2, mitochondrial	0.87974564	0.0185476
Q6P069	Sorcin	0.87267664	0.00318565
Q9ERS2	NADH dehydrogenase [ubiquinone] 1 alpha subcomplex subunit 13	0.86688929	0.00590581
Q91WS0	CDGSH iron-sulfur domain-containing protein 1	0.86302742	0.03406038
P14094	Sodium/potassium-transporting ATPase subunit beta-1	0.86187688	0.02391984
Q7TMF3	NADH dehydrogenase [ubiquinone] 1 alpha subcomplex subunit 12	0.8615585	0.03845908
Q9D7G0	Ribose-phosphate pyrophosphokinase 1	0.85929152	0.02159006
P60904	DnaJ homolog subfamily C member 5	0.85215533	0.00183989
O08788	Dynactin subunit 1	0.85102698	0.00851758
Q91YT0	NADH dehydrogenase [ubiquinone] flavoprotein 1, mitochondrial	0.85083051	0.01363154
Q8QZS1	3-hydroxyisobutyryl-CoA hydrolase, mitochondrial	0.84804248	0.00924658
Q6PIC6	Sodium/potassium-transporting ATPase subunit alpha-3	0.8480239	0.02793432
Q9JIS5	Synaptic vesicle glycoprotein 2A	0.84364041	0.00352979
P63037	DnaJ homolog subfamily A member 1	0.8302141	0.03580565
Q5U458	DnaJ homolog subfamily C member 11	0.82983573	0.0247101
Q99JP7	Glutathione hydrolase 7	0.82905659	0.03224608
Q8BHC1	Ras-related protein Rab-39B	0.81886045	0.03633878
Q91VN4	MICOS complex subunit Mic25	0.81444019	0.02699587
Q7TT37	Elongator complex protein 1	0.81044485	0.04836832
Q06890	Clusterin	0.79903438	0.04559052
P09528	Ferritin heavy chain	0.79080916	0.0192747
O88545	COP9 signalosome complex subunit 6	0.78891648	0.0264992
Q61418	H(+)/Cl(-) exchange transporter 4	0.78617904	0.02495443
Q64436	Potassium-transporting ATPase alpha chain 1	0.779414	0.01002401
P61264	Syntaxin-1B	0.77507592	0.01218806
O70228	Probable phospholipid-transporting ATPase IIA	0.7610778	0.04815936
P47740	Aldehyde dehydrogenase family 3 member A2	0.75759204	0.03504264
Q8VEB4	Group XV phospholipase A2	0.75398394	0.0275253
Q60870	Receptor expression-enhancing protein 5	0.74632932	0.02288104
Q3UJU9	Regulator of microtubule dynamics protein 3	0.74577951	0.0439637
Q61712	DnaJ homolog subfamily C member 1	0.73137297	0.04270055
P11627	Neural cell adhesion molecule L1	0.73016156	0.02944691
Q9QUJ7	Long-chain-fatty-acid--CoA ligase 4	0.72831787	0.02765463
P01887	Beta-2-microglobulin	0.72584643	0.04301478
O89079	Coatomer subunit epsilon	0.71292752	0.03890297
Q9QYA2	Mitochondrial import receptor subunit TOM40 homolog	0.70951217	0.03298886
Q9WV85	Nucleoside diphosphate kinase 3	0.70654258	0.00171499

Accession Number	Protein Name	ratio wt bgc vs. wt bgi	p value
A2ADY9	Protein DDI1 homolog 2	0.70646992	0.03451381
P26645	Myristoylated alanine-rich C-kinase substrate	0.68724007	0.03873673
Q91WG4	Elongator complex protein 2	0.67916466	0.04259465
P05366	Serum amyloid A-1 protein	0.66263229	0.04094214
Q8VBT0	Thioredoxin-related transmembrane protein 1	0.65859337	0.00140669
Q9QZI8	Serine incorporator 1	0.65791877	0.04224245
Q02013	Aquaporin-1	0.65178321	0.00028787
O89051	Integral membrane protein 2B	0.63480852	0.04497524
Q8BM85	TBC domain-containing protein kinase-like protein	0.59965443	0.0196957
Q8BMI3	ADP-ribosylation factor-binding protein GGA3	0.5993644	0.02818664
Q8CFE4	SCY1-like protein 2	0.59391321	0.02398177
Q91ZU6	Dystonin	0.58721289	0.025561
Q8BYN5	FSD1-like protein	0.57594039	0.02910675
P31750	RAC-alpha serine/threonine-protein kinase	0.57031202	0.04193927
Q9D554	Splicing factor 3A subunit 3	0.56856704	0.02987042
O35658	Complement component 1 Q subcomponent-binding protein, mitochondrial	0.55731983	0.04777864
P83887	Tubulin gamma-1 chain	0.53944623	0.00652253
P01027	Complement C3	0.53225332	0.01871014
Q9D020	Cytosolic 5'-nucleotidase 3A	0.52662245	0.02254
O70503	Very-long-chain 3-oxoacyl-CoA reductase	0.52257417	0.01262309
Q921I1	Serotransferrin	0.52019894	0.03302309
A6X935	Inter alpha-trypsin inhibitor, heavy chain 4	0.51660467	0.02812628
Q8C7D2	Protein cereblon	0.50478292	0.00282135
Q9JI19	Acidic fibroblast growth factor intracellular-binding protein	0.49750489	0.00444943
Q9D9V3	Ethylmalonyl-CoA decarboxylase	0.40362973	0.03870626
Q3UYG8	ADP-ribose glycohydrolase MACROD2	0.39077281	0.0468278
Q91WP6	Serine protease inhibitor A3N	0.35192974	0.02490918
O09172	Glutamate--cysteine ligase regulatory subunit	0.33350262	0.00170627
Q3UHG7	DENN domain-containing protein 11	0.30385959	0.01399564
P14602	Heat shock protein beta-1	0.28735041	0.04653098
Q99JH7	Calsyntenin-3	0.24718812	0.04145939

Table S1

Significantly regulated proteins in wt bgc vs. wt bgi.

Mass spectrometric analyses of contralateral basal ganglia (bgc; n = 4) and ipsilateral basal ganglia (bgi; n = 5) of wild-type (wt) mice 24 h after transient middle cerebral artery occlusion. The table indicates the protein accession number, protein name, ratio and p-values; n-numbers

represent samples of individual mice. For statistical analysis unpaired Students t-test was applied.

Table S2

Accession Number	Protein Name	ratio wt bgc vs. RKIP^{wt} bgi	p value
Q8R3Z5	Voltage-dependent L-type calcium channel subunit beta-1	4.91947131	0.00296349
Q99J45	Nuclear receptor-binding protein	4.34731532	0.01245639
Q8BGU5	Cyclin-Y	4.08114439	0.00462016
P0C7L0	WAS/WASL-interacting protein family member 3	3.7771288	0.02129021
Q3UTQ8	Cyclin-dependent kinase-like 5	3.57352733	0.0260915
Q68ED7	CREB-regulated transcription coactivator 1	3.56957787	0.00014716
Q3UHD1	Adhesion G protein-coupled receptor B1	3.10953868	0.00267717
Q9Z275	Retinaldehyde-binding protein 1	3.0054013	0.02351401
Q9ERT9	Protein phosphatase 1 regulatory subunit 1A	2.97990686	0.00603768
O35345	Importin subunit alpha-7	2.9336552	0.00674457
Q9JHQ5	Leucine zipper transcription factor-like protein 1	2.83938379	0.00063958
P50114	Protein S100-B	2.68578608	0.01713028
P17665	Cytochrome c oxidase subunit 7C, mitochondrial	2.64889435	0.04010896
Q8R123	FAD synthase	2.49513577	0.0305477
Q04750	DNA topoisomerase 1	2.45500221	0.00832458
Q8VCN9	Tubulin-specific chaperone C	2.44799954	0.00338602
A2APV2	Formin-like protein 2	2.44531422	0.02181284
Q810A7	ATP-dependent RNA helicase DDX42	2.43878798	0.03748901
P26048	Gamma-aminobutyric acid receptor subunit alpha-2	2.39190331	0.02718495
Q922J3	CAP-Gly domain-containing linker protein 1	2.3692002	0.01723125
P20065	Thymosin beta-4	2.36135859	0.04599933
Q5U3K5	Rab-like protein 6	2.34936109	0.03063621
D3YUB6	BTB/POZ domain-containing protein 8	2.32506959	0.04612715
Q91WT9	Cystathionine beta-synthase	2.31712417	0.0354883
Q9Z2V5	Histone deacetylase 6	2.30544233	0.02842032
Q9JKY5	Huntingtin-interacting protein 1-related protein	2.28446395	0.00127447
Q7TSC1	Protein PRRC2A	2.2597776	0.01568357
P26231	Catenin alpha-1	2.24710241	0.02081809
Q91ZP9	N-terminal EF-hand calcium-binding protein 2	2.22120475	0.04977671
Q80WC7	Arf-GAP domain and FG repeat-containing protein 2	2.22109066	0.01094822
P62309	Small nuclear ribonucleoprotein G	2.21718669	0.04324232
P63154	Crooked neck-like protein 1	2.20765873	0.03036787
Q9CRC9	Glucosamine-6-phosphate isomerase 2	2.18658528	0.04720766
Q64152	Transcription factor BTF3	2.1469165	0.03116976
Q5SV85	Synergin gamma	2.12073952	0.01095109
Q9ESZ8	General transcription factor II-I	2.08147137	0.04582155
Q9CQW0	ER membrane protein complex subunit 6	2.06596872	0.02747128

Accession Number	Protein Name	ratio wt bgc vs. RKIP^{wt} bgi	p value
Q6PE13	Proline-rich transmembrane protein 3	2.04623303	0.039591
Q9D2R6	Cytochrome c oxidase assembly factor 3 homolog, mitochondrial	2.04462905	0.04784103
Q64378	Peptidyl-prolyl cis-trans isomerase FKBP5	2.04190745	0.03884174
Q9R1P3	Proteasome subunit beta type-2	2.03130683	0.01359709
Q69ZW3	EH domain-binding protein 1	1.99550626	0.01155797
Q69ZR2	E3 ubiquitin-protein ligase HECTD1	1.97772326	0.04534061
Q8VI75	Importin-4	1.96770694	0.04176691
Q9CZ28	Vacuolar-sorting protein SNF8	1.94769253	0.02078016
P84086	Complexin-2	1.93639205	0.04423464
Q3UX10	Tubulin alpha chain-like 3	1.89277777	0.0085239
Q3V3Q7	Phosphofurin acidic cluster sorting protein 2	1.8866851	0.0184932
Q6PGL7	WASH complex subunit 2	1.88100891	0.04751164
P83510	Traf2 and NCK-interacting protein kinase	1.87784361	0.00584251
Q3TBW2	39S ribosomal protein L10, mitochondrial	1.87619433	0.02339781
Q8R1F5	Putative hydroxypyruvate isomerase	1.85659866	0.02494653
Q8BRF7	Sec1 family domain-containing protein 1	1.83205661	0.02886913
P99027	60S acidic ribosomal protein P2	1.82837659	0.00373707
Q8BG40	Katanin p80 WD40 repeat-containing subunit B1	1.82543749	0.01365686
Q9JHL1	Na(+)/H(+) exchange regulatory cofactor NHE-RF2	1.80717185	0.00957108
Q80X50	Ubiquitin-associated protein 2-like	1.80401939	0.02739685
Q9CQD4	Charged multivesicular body protein 1b-2	1.79339968	0.00977237
Q9QZQ8	Core histone macro-H2A.1	1.79327747	0.03233265
A2BDX3	Adenylyltransferase and sulfurtransferase MOCS3	1.79251189	0.0184223
Q9DBR7	Protein phosphatase 1 regulatory subunit 12A	1.75806998	0.0078278
Q91V76	Ester hydrolase C11orf54 homolog	1.75275177	0.03746536
Q8R0H9	ADP-ribosylation factor-binding protein GGA1	1.74536788	0.04790424
P49442	Inositol polyphosphate 1-phosphatase	1.73657401	0.01791839
Q9D5V6	Synapse-associated protein 1	1.72655323	0.01087709
Q9WU28	Prefoldin subunit 5	1.70376465	0.04403511
Q9R1Q9	V-type proton ATPase subunit S1	1.70158502	0.02426032
Q60714	Long-chain fatty acid transport protein 1	1.68504134	0.04196721
Q8CHU3	Epsin-2	1.68243007	0.00683973
Q6P9R2	Serine/threonine-protein kinase OSR1	1.65835926	0.03576708
Q9DB10	Essential MCU regulator, mitochondrial	1.65566587	0.0433803
Q8CI71	Syndetin	1.65564827	0.03523027
P39087	Glutamate receptor ionotropic, kainate 2	1.6511872	0.01823531
Q3UBX0	Transmembrane protein 109	1.62665813	0.01409129
Q3UZP4	Small VCP/p97-interacting protein	1.62569321	0.03865517
Q8VI63	MOB kinase activator 2	1.62450464	0.02639489
O88745	Scrapie-responsive protein 1	1.60828235	0.01001853

Accession Number	Protein Name	ratio wt bgc vs. RKIP^{wt} bgi	p value
P47754	F-actin-capping protein subunit alpha-2	1.56582796	0.02809059
Q58A65	C-Jun-amino-terminal kinase-interacting protein 4	1.55905801	0.01072911
P54923	[Protein ADP-ribosylarginine] hydrolase	1.55114631	0.01212608
Q6P4T2	U5 small nuclear ribonucleoprotein 200 kDa helicase	1.54225101	0.03723063
Q9D0J8	Parathymosin	1.54013774	0.0341005
Q8BWY3	Eukaryotic peptide chain release factor subunit 1	1.53345811	0.02396221
Q91V36	Nuclear receptor-binding protein 2	1.52983702	0.04451457
Q01063	cAMP-specific 3',5'-cyclic phosphodiesterase 4D	1.52935803	0.02952958
Q8CFI0	E3 ubiquitin-protein ligase NEDD4-like	1.52802988	0.02318914
Q3UHB8	Coiled-coil domain-containing protein 177	1.507793	0.03743559
Q9D7V2	LysM and putative peptidoglycan-binding domain-containing protein 2	1.50379182	0.02320928
Q3UEB3	Poly(U)-binding-splicing factor PUF60	1.50341442	0.02530086
Q91XD6	Vacuolar protein-sorting-associated protein 36	1.50157569	0.00079793
Q9QYB5	Gamma-adducin	1.49269475	0.04279331
P28028	Serine/threonine-protein kinase B-raf	1.48146893	0.02604726
Q8C052	Microtubule-associated protein 1S	1.4660854	0.0010366
Q9WV69	Dematin	1.4633326	0.02525481
Q8C8N2	Protein SCAI	1.45361741	0.01807973
Q6PFQ7	Ras GTPase-activating protein 4	1.44802842	0.00861973
Q99JY8	Phospholipid phosphatase 3	1.42352407	0.04058585
Q810B6	Rabankyrin-5	1.41510973	0.02844752
Q9EQZ6	Rap guanine nucleotide exchange factor 4	1.41478483	0.01711894
P97797	Tyrosine-protein phosphatase non-receptor type substrate 1	1.40530663	0.02259358
O35280	Serine/threonine-protein kinase Chk1	1.40325264	0.0060024
Q6P8X1	Sorting nexin-6	1.38742902	0.00878041
Q7TQD2	Tubulin polymerization-promoting protein	1.38113481	0.00998524
Q8BNY6	Neuronal calcium sensor 1	1.37382174	0.02708851
Q9QYC0	Alpha-adducin	1.37330184	0.01244665
P62962	Profilin-1	1.37004398	0.01573577
Q8CCN5	Breast carcinoma-amplified sequence 3 homolog	1.36069012	0.0411325
Q9EPL8	Importin-7	1.35910576	0.01738084
Q80YA3	Phospholipase DDHD1	1.35510187	0.04971989
Q8VE22	28S ribosomal protein S23, mitochondrial	1.35498501	0.00798755
Q9R257	Heme-binding protein 1	1.35054916	0.00090423
Q9DAS9	Guanine nucleotide-binding protein G(I)/G(S)/G(O) subunit gamma-12	1.34691589	0.04496255
Q8K2K6	Arf-GAP domain and FG repeat-containing protein 1	1.34673966	0.0474337
Q91YR1	Twinfilin-1	1.34499376	0.0379611

Accession Number	Protein Name	ratio wt bgc vs. RKIP^{wt} bgi	p value
Q9CQJ6	Density-regulated protein	1.3399979	0.00427897
P40336	Vacuolar protein sorting-associated protein 26A	1.33101243	0.04863424
P63325	40S ribosomal protein S10	1.31102891	0.04841968
Q9D5V5	Cullin-5	1.30870334	0.04102665
P51859	Hepatoma-derived growth factor	1.3026939	0.03283007
Q62189	U1 small nuclear ribonucleoprotein A	1.30084013	0.04631289
Q99KI3	ER membrane protein complex subunit 3	1.29895521	0.0089189
Q9CR95	Adaptin ear-binding coat-associated protein 1	1.29396903	0.01391342
P21550	Beta-enolase	1.27966595	0.02082816
Q8BY89	Choline transporter-like protein 2	1.27789572	0.02845513
Q5HZI9	Solute carrier family 25 member 51	1.27437942	0.00043265
Q9D173	Mitochondrial import receptor subunit TOM7 homolog	1.27294684	0.02865488
P62743	AP-2 complex subunit sigma	1.27261627	0.01902926
P63242	Eukaryotic translation initiation factor 5A-1	1.27256695	0.0418308
Q61598	Rab GDP dissociation inhibitor beta	1.26914033	0.0410113
Q8VD37	SH3-containing GRB2-like protein 3-interacting protein 1	1.26123921	0.00137319
Q91YJ2	Sorting nexin-4	1.25481835	0.00375028
Q9D6F9	Tubulin beta-4A chain	1.24621467	0.00389639
Q9CY58	Plasminogen activator inhibitor 1 RNA-binding protein	1.23946704	0.00578433
Q9Z0P4	Paralemmin-1	1.23753237	0.04787388
Q64010	Adapter molecule crk	1.23700974	0.0411499
P10126	Elongation factor 1-alpha 1	1.23623615	0.02148852
P26883	Peptidyl-prolyl cis-trans isomerase FKBP1A	1.23589662	0.02933068
Q3UHD6	Sorting nexin-27	1.22865622	0.01480403
Q61171	Peroxiredoxin-2	1.21961858	0.02476354
P57746	V-type proton ATPase subunit D	1.21785112	0.02395858
Q9D2N4	Dystrobrevin alpha	1.21557924	0.01712681
Q99L45	Eukaryotic translation initiation factor 2 subunit 2	1.1969793	0.00459652
Q922H2	[Pyruvate dehydrogenase (acetyl-transferring)] kinase isozyme 3, mitochondrial	1.193404	0.0056321
Q3UHX2	28 kDa heat- and acid-stable phosphoprotein	1.19216169	0.0400838
Q91ZJ5	UTP--glucose-1-phosphate uridylyltransferase	1.17642905	0.02827367
P70288	Histone deacetylase 2	1.16399614	0.03006749
Q9CZY3	Ubiquitin-conjugating enzyme E2 variant 1	1.13392304	0.02235511
Q8BK08	Transmembrane protein 11, mitochondrial	1.13183831	0.04994428
Q8C1A5	Thimet oligopeptidase	1.11063693	0.03334593
Q8R1Q8	Cytoplasmic dynein 1 light intermediate chain 1	1.10521535	0.02700646
P63321	Ras-related protein Ral-A	0.9195068	0.04511547
P35486	Pyruvate dehydrogenase E1 component subunit alpha, somatic form, mitochondrial	0.90750777	0.00252662

Accession Number	Protein Name	ratio wt bgc vs. RKIP^{wt} bgi	p value
P11983	T-complex protein 1 subunit alpha	0.90166973	0.02256089
Q99LY9	NADH dehydrogenase [ubiquinone] iron-sulfur protein 5	0.89108363	0.01212549
Q9CQ69	Cytochrome b-c1 complex subunit 8	0.87565344	0.02275804
P63017	Heat shock cognate 71 kDa protein	0.87217394	0.01017752
Q9JII5	DAZ-associated protein 1	0.86467183	0.02839797
Q9DB77	Cytochrome b-c1 complex subunit 2, mitochondrial	0.85298453	0.00502217
O08788	Dynactin subunit 1	0.85197757	0.00886363
Q9JHU4	Cytoplasmic dynein 1 heavy chain 1	0.85065379	0.03948187
Q9D394	Protein RUFY3	0.82999468	0.04844227
P14685	26S proteasome non-ATPase regulatory subunit 3	0.82645518	0.02249015
P14148	60S ribosomal protein L7	0.82177921	0.04971009
Q9CR67	Transmembrane protein 33	0.81188179	0.01487289
P70296	Phosphatidylethanolamine-binding protein 1	0.81086193	0.00010226
Q91YT0	NADH dehydrogenase [ubiquinone] flavoprotein 1, mitochondrial	0.809134	0.0002014
Q9DCX2	ATP synthase subunit d, mitochondrial	0.80745001	0.02704297
Q6ZWU9	40S ribosomal protein S27	0.80398244	0.04585383
Q9Z0G0	PDZ domain-containing protein GIPC1	0.80137292	0.02636172
P56391	Cytochrome c oxidase subunit 6B1	0.80113151	0.0451912
O88543	COP9 signalosome complex subunit 3	0.79910609	0.03097421
Q9WV85	Nucleoside diphosphate kinase 3	0.79410915	0.01008921
P43276	Histone H1.5	0.79326046	0.02984911
Q64520	Guanylate kinase	0.78226594	0.01746512
O54901	OX-2 membrane glycoprotein	0.77313278	0.02633984
P62717	60S ribosomal protein L18a	0.76615165	0.03054727
P63037	DnaJ homolog subfamily A member 1	0.76404805	0.02775026
P33173	Kinesin-like protein KIF1A	0.76398792	0.03895258
Q9CQQ7	ATP synthase F(0) complex subunit B1, mitochondrial	0.76075294	0.02611363
Q3UIU2	NADH dehydrogenase [ubiquinone] 1 beta subcomplex subunit 6	0.75603631	0.02404477
Q8R361	Rab11 family-interacting protein 5	0.75534726	0.02350897
Q3UVK0	Endoplasmic reticulum metallopeptidase 1	0.75449927	0.00136737
Q8K1J6	CCA tRNA nucleotidyltransferase 1, mitochondrial	0.75039229	0.02538885
P26645	Myristoylated alanine-rich C-kinase substrate	0.74521766	0.01893671
P27659	60S ribosomal protein L3	0.74219034	0.01913142
Q8BMJ3	Eukaryotic translation initiation factor 1A, X-chromosomal	0.73365349	0.04840934
O88507	Ciliary neurotrophic factor receptor subunit alpha	0.72510074	0.00238751
Q9D710	Thioredoxin-related transmembrane protein 2	0.72177022	0.00760601
Q8VBV7	COP9 signalosome complex subunit 8	0.71802604	0.02331722

Accession Number	Protein Name	ratio wt bgc vs. RKIP^{wt} bgi	p value
Q80ZS3	28S ribosomal protein S26, mitochondrial	0.71574672	0.04966013
Q9EQP2	EH domain-containing protein 4	0.71165578	0.0335318
Q9Z1P6	NADH dehydrogenase [ubiquinone] 1 alpha subcomplex subunit 7	0.71159621	0.04989322
Q9WUM4	Coronin-1C	0.70938347	0.00836346
Q8BYN5	FSD1-like protein	0.69929794	0.03781566
Q9CZB0	Succinate dehydrogenase cytochrome b560 subunit, mitochondrial	0.69923703	0.02926353
P62983	Ubiquitin-40S ribosomal protein S27a	0.69749678	0.01169956
Q9ES89	Exostosin-like 2	0.69681696	0.03497378
Q9DCS9	NADH dehydrogenase [ubiquinone] 1 beta subcomplex subunit 10	0.67631251	0.00353033
Q3UE37	Ubiquitin-conjugating enzyme E2 Z	0.67174898	0.00659857
P23492	Purine nucleoside phosphorylase	0.66725845	0.0425326
P35564	Calnexin	0.66190199	0.02320024
Q9DCS3	Enoyl-[acyl-carrier-protein] reductase, mitochondrial	0.66093223	0.02880646
O35969	Guanidinoacetate N-methyltransferase	0.64481385	0.01770397
O55026	Ectonucleoside triphosphate diphosphohydrolase 2	0.63088594	0.03156237
P13020	Gelsolin	0.63011646	0.00300681
Q7TNC4	Putative RNA-binding protein Luc7-like 2	0.62889744	0.03927182
Q61712	DnaJ homolog subfamily C member 1	0.62262327	0.00734666
Q9CQJ8	NADH dehydrogenase [ubiquinone] 1 beta subcomplex subunit 9	0.61338227	0.00661208
Q8BMI3	ADP-ribosylation factor-binding protein GGA3	0.60822603	0.02488495
O35658	Complement component 1 Q subcomponent-binding protein, mitochondrial	0.60384744	0.02838291
Q61335	B-cell receptor-associated protein 31	0.5922582	0.00813851
Q60870	Receptor expression-enhancing protein 5	0.59074143	0.0007251
Q6VNS1	NT-3 growth factor receptor	0.58595943	0.00021373
Q8BYI8	Protein FAM234B	0.58332614	0.04521641
P03911	NADH-ubiquinone oxidoreductase chain 4	0.58125266	0.03792601
Q8BT07	Centrosomal protein of 55 kDa	0.57745004	0.02877833
P00158	Cytochrome b	0.55080872	0.00643556
Q9CQ91	NADH dehydrogenase [ubiquinone] 1 alpha subcomplex subunit 3	0.55011215	0.00207791
Q8BSL7	ADP-ribosylation factor 2	0.55006617	0.04564284
E9PV24	Fibrinogen alpha chain	0.51142159	0.00578888
P56213	FAD-linked sulfhydryl oxidase ALR	0.49949359	0.04216582
Q91X72	Hemopexin	0.48148041	0.0023615
O35963	Ras-related protein Rab-33B	0.46414933	0.03063231
Q8K0E8	Fibrinogen beta chain	0.45826791	0.00214426
Q8VCM7	Fibrinogen gamma chain	0.43818855	0.01286295
Q3URS9	Coiled-coil domain-containing protein 51	0.43048249	0.0150508

Accession Number	Protein Name	ratio wt bgc vs. RKIP^{wt} bgi	p value
Q9CZX0	Elongator complex protein 3	0.42817878	0.03129178
O35683	NADH dehydrogenase [ubiquinone] 1 alpha subcomplex subunit 1	0.42111999	0.00946214
Q8R4V2	Dual specificity protein phosphatase 15	0.4207971	0.02538121
P56375	Acylphosphatase-2	0.40147295	0.00323521
Q91WP6	Serine protease inhibitor A3N	0.38539963	0.03174061
P11276	Fibronectin	0.37903259	0.03998057
Q3UVV9	von Willebrand factor A domain-containing protein 3A	0.37838542	0.01496756
Q3UYG8	ADP-ribose glycohydrolase MACROD2	0.32337429	0.02165985
Q61646	Haptoglobin	0.3154839	0.04618389

Table S2

Significantly regulated proteins in wt bgc vs. RKIP^{wt} bgi.

Mass spectrometric analyses of contralateral basal ganglia (bgc) of wild-type (wt; n = 4) mice and ipsilateral basal ganglia (bgi) of mice ubiquitously overexpressing RKIP^{wt} (RKIP^{wt}; n = 2) 24 h after transient middle cerebral artery occlusion. The table indicates the protein accession number, protein name, ratio and p-values; n-numbers represent samples of individual mice. For statistical analysis unpaired Students t-test was applied.

Table S3

Accession Number	Protein Name	ratio wt bgc vs. RKIP^{S153A} bgi	p value
P24529	Tyrosine 3-monooxygenase	11.0274471	0.0346985
Q8BXT1	Regulator of G-protein signaling 8	9.18914066	0.01031413
P09470	Angiotensin-converting enzyme	3.76751553	0.03527443
Q8R3Q6	Coiled-coil domain-containing protein 58	3.47007986	0.03694731
P55096	ATP-binding cassette sub-family D member 3	3.29264981	0.00278938
Q04750	DNA topoisomerase 1	3.02800725	0.00349481
Q91ZP9	N-terminal EF-hand calcium-binding protein 2	2.76555268	0.01595864
Q8R3Z5	Voltage-dependent L-type calcium channel subunit beta-1	2.74941954	0.02887476
O35393	Ephrin-B3	2.67813189	0.00081378
Q9CRC9	Glucosamine-6-phosphate isomerase 2	2.58315894	0.03632389
Q9QX60	Deoxyguanosine kinase, mitochondrial	2.51406211	0.00497979
Q80WC7	Arf-GAP domain and FG repeat-containing protein 2	2.47364602	0.00959413
Q69ZR2	E3 ubiquitin-protein ligase HECTD1	2.39858018	0.0265301
Q5EG47	5'-AMP-activated protein kinase catalytic subunit alpha-1	2.25194972	0.00395299
Q924C5	Alpha-protein kinase 3	2.20506602	0.01741466
Q9D2R6	Cytochrome c oxidase assembly factor 3 homolog, mitochondrial	2.14070677	0.03779122
P0C7L0	WAS/WASL-interacting protein family member 3	2.12947746	0.04330945
Q3TBW2	39S ribosomal protein L10, mitochondrial	2.00276309	0.02314544
Q80TE7	Leucine-rich repeat-containing protein 7	1.99785455	0.04869389
O35387	HCLS1-associated protein X-1	1.97042262	0.02664575
P63154	Crooked neck-like protein 1	1.96135981	0.03568309
Q99LS3	Phosphoserine phosphatase	1.96014213	0.01929947
Q62084	Protein phosphatase 1 regulatory subunit 14B	1.94994973	0.04072599
Q9DC07	LIM zinc-binding domain-containing Nebulette	1.94587402	0.0145834
Q921Q7	Ras and Rab interactor 1	1.92947958	0.03293865
Q7TQH0	Ataxin-2-like protein	1.92720107	0.01246134
Q9CQ80	Vacuolar protein-sorting-associated protein 25	1.82810506	0.02955241
Q8CE90	Dual specificity mitogen-activated protein kinase kinase 7	1.76310912	0.04583045
Q9CQI7	U2 small nuclear ribonucleoprotein B''	1.76290428	0.01575334
Q3TYX3	SET and MYND domain-containing protein 5	1.74701562	0.04950172
Q8R0H9	ADP-ribosylation factor-binding protein GGA1	1.72947558	0.0027187
Q08943	FACT complex subunit SSRP1	1.71647505	0.02452814
Q9DCF9	Translocon-associated protein subunit gamma	1.70635754	0.0329452
Q9DB10	Essential MCU regulator, mitochondrial	1.68079155	0.01095941
Q80XK6	Autophagy-related protein 2 homolog B	1.64760811	0.03746632
Q6NZB0	DnaJ homolog subfamily C member 8	1.63284628	0.01255392

Accession Number	Protein Name	ratio wt bgc vs. RKIP^{S153A} bgi	p value
Q3UHL1	CaM kinase-like vesicle-associated protein	1.59661298	0.02906372
Q8R1F5	Putative hydroxypyruvate isomerase	1.58952763	0.02169667
Q3V3Q7	Phosphofurin acidic cluster sorting protein 2	1.58063427	0.01259528
Q6ZQ82	Rho GTPase-activating protein 26	1.57822439	0.02358919
P70297	Signal transducing adapter molecule 1	1.53948842	0.01920886
P47746	Cannabinoid receptor 1	1.5390548	0.03964898
Q8BWY3	Eukaryotic peptide chain release factor subunit 1	1.51130338	0.01886849
Q8BY87	Ubiquitin carboxyl-terminal hydrolase 47	1.50850527	0.01314965
Q8VHK5	Membrane protein MLC1	1.50523625	0.02402014
Q8BH69	Selenide, water dikinase 1	1.48227829	0.04164836
Q8K0G5	EARP and GARP complex-interacting protein 1	1.48142075	0.01442199
Q8BZF8	Phosphoglucomutase-like protein 5	1.47597545	0.00150178
Q9JLB0	MAGUK p55 subfamily member 6	1.45434854	0.02101165
B9EJA2	Cortactin-binding protein 2	1.44589488	0.03557802
Q9CYN9	Renin receptor	1.44516233	0.0125143
Q9CQD4	Charged multivesicular body protein 1b-2	1.44400973	0.03612839
Q9D7X8	Gamma-glutamylcyclotransferase	1.43999154	0.01309952
Q9QWR8	Alpha-N-acetylgalactosaminidase	1.43780964	0.04089665
Q91YR1	Twinfilin-1	1.43103763	0.02263615
P20352	Tissue factor	1.42418055	0.04798224
P62880	Guanine nucleotide-binding protein G(I)/G(S)/G(T) subunit beta-2	1.42266278	0.02231093
P28352	DNA-(apurinic or apyrimidinic site) lyase	1.42034722	0.03587361
A2A8U2	Transmembrane protein 201	1.39733435	0.0288043
Q9CWU6	Ubiquinol-cytochrome-c reductase complex assembly factor 1	1.39019686	0.02960174
Q9ES28	Rho guanine nucleotide exchange factor 7	1.39006473	0.03892696
Q8BG67	Protein EFR3 homolog A	1.38974596	0.03163375
Q8K3G9	DCC-interacting protein 13-beta	1.38521981	0.04559342
Q9CZR3	Mitochondrial import receptor subunit TOM40B	1.3764042	0.02331215
P70288	Histone deacetylase 2	1.36802057	0.01706701
Q9R1Q9	V-type proton ATPase subunit S1	1.35966408	0.02941595
P62962	Profilin-1	1.34684735	0.01443264
Q60738	Zinc transporter 1	1.33305196	0.01400073
Q6TL19	Guanylate cyclase 2G	1.32405865	0.02595951
Q9JKB1	Ubiquitin carboxyl-terminal hydrolase isozyme L3	1.31470201	0.03405431
Q8VE22	28S ribosomal protein S23, mitochondrial	1.31395035	0.04045502
Q8VHI6	Wiskott-Aldrich syndrome protein family member 3	1.31165402	0.04623465
Q9QYG0	Protein NDRG2	1.3075406	0.00337451
Q64737	Trifunctional purine biosynthetic protein adenosine-3	1.30720363	0.0366887

Accession Number	Protein Name	ratio wt bgc vs. RKIP^{S153A} bgi	p value
P52623	Uridine-cytidine kinase 1	1.29918673	0.03208081
P61087	Ubiquitin-conjugating enzyme E2 K	1.2833411	0.02315297
Q8C052	Microtubule-associated protein 1S	1.27580698	0.03375737
O35864	COP9 signalosome complex subunit 5	1.26308323	0.04341213
Q922H2	[Pyruvate dehydrogenase (acetyl-transferring)] kinase isozyme 3, mitochondrial	1.23865473	0.00263863
P26883	Peptidyl-prolyl cis-trans isomerase FKBP1A	1.23769505	0.03458507
Q8BFY6	Peflin	1.23499506	0.04117044
Q9WV80	Sorting nexin-1	1.22820892	0.04801509
P48774	Glutathione S-transferase Mu 5	1.22684563	0.03453433
P04925	Major prion protein	1.22431635	0.02892349
P10126	Elongation factor 1-alpha 1	1.21785019	0.04460603
Q91XD6	Vacuolar protein-sorting-associated protein 36	1.21549772	0.00409244
P62141	Serine/threonine-protein phosphatase PP1-beta catalytic subunit	1.21017021	0.00199046
O35621	Phosphomannomutase 1	1.20204888	0.04013906
Q9JHK4	Geranylgeranyl transferase type-2 subunit alpha	1.19145459	0.0299511
Q9CQJ6	Density-regulated protein	1.19125236	0.02866956
Q8K021	Secretory carrier-associated membrane protein 1	1.18522963	0.02486887
Q76MZ3	Serine/threonine-protein phosphatase 2A 65 kDa regulatory subunit A alpha isoform	1.18487615	0.03105606
Q9QUM9	Proteasome subunit alpha type-6	1.17824783	0.00845391
Q91ZJ5	UTP--glucose-1-phosphate uridylyltransferase	1.16110804	0.03938932
P61027	Ras-related protein Rab-10	1.1518999	0.04209961
P48962	ADP/ATP translocase 1	1.15142369	0.04298689
Q8CGK3	Lon protease homolog, mitochondrial	1.15124504	0.02671386
Q9WTN0	Geranylgeranyl pyrophosphate synthase	1.14514619	0.03091078
O55234	Proteasome subunit beta type-5	1.14160949	0.0345465
P16858	Glyceraldehyde-3-phosphate dehydrogenase	1.1196384	0.01996177
Q8R1Q8	Cytoplasmic dynein 1 light intermediate chain 1	1.11810822	0.02943493
Q8BH59	Calcium-binding mitochondrial carrier protein Aralar1	1.10450211	0.04798978
P60904	DnaJ homolog subfamily C member 5	1.10055317	0.03602822
P56371	Ras-related protein Rab-4A	1.0861934	0.04998343
Q9Z1S5	Neuronal-specific septin-3	0.90002855	0.04229255
Q8VEH3	ADP-ribosylation factor-like protein 8A	0.88839284	0.02800982
Q9D6J6	NADH dehydrogenase [ubiquinone] flavoprotein 2, mitochondrial	0.87697081	0.0232016
P11983	T-complex protein 1 subunit alpha	0.86969856	0.01940567
Q80XI4	Phosphatidylinositol 5-phosphate 4-kinase type-2 beta	0.85448429	0.01575744
Q8BGQ7	Alanine--tRNA ligase, cytoplasmic	0.85206333	0.03443107
Q8CHH9	Septin-8	0.84974605	0.01100054

Accession Number	Protein Name	ratio wt bgc vs. RKIP^{S153A} bgi	p value
Q9JHU4	Cytoplasmic dynein 1 heavy chain 1	0.84634783	0.00912831
O35857	Mitochondrial import inner membrane translocase subunit TIM44	0.83896984	0.01324644
P14094	Sodium/potassium-transporting ATPase subunit beta-1	0.8363704	0.00987516
Q61644	Protein kinase C and casein kinase substrate in neurons protein 1	0.83370619	0.00569164
O09111	NADH dehydrogenase [ubiquinone] 1 beta subcomplex subunit 11, mitochondrial	0.82806812	0.04769368
O08547	Vesicle-trafficking protein SEC22b	0.82560996	0.03828569
Q9EP69	Phosphatidylinositide phosphatase SAC1	0.82541332	0.01588701
Q9CR57	60S ribosomal protein L14	0.81742956	0.0421377
O70228	Probable phospholipid-transporting ATPase IIA	0.81707175	0.0313572
Q9CR67	Transmembrane protein 33	0.80985573	0.01310429
O08788	Dynactin subunit 1	0.80964648	0.00796467
P12367	cAMP-dependent protein kinase type II-alpha regulatory subunit	0.80171584	0.04070144
P61079	Ubiquitin-conjugating enzyme E2 D3	0.79493439	0.00406477
Q8BP71	RNA binding protein fox-1 homolog 2	0.7892865	0.02822183
P41105	60S ribosomal protein L28	0.7870759	0.04030345
O35295	Transcriptional activator protein Pur-beta	0.78684507	0.04897322
P35980	60S ribosomal protein L18	0.78372225	0.0369201
P47911	60S ribosomal protein L6	0.78363891	0.02647164
Q6NZL0	Protein SOGA3	0.78160913	0.00215616
P00920	Carbonic anhydrase 2	0.77938955	0.02032205
Q9D023	Mitochondrial pyruvate carrier 2	0.77872308	0.02416586
P62918	60S ribosomal protein L8	0.76691982	0.00630734
Q91XV3	Brain acid soluble protein 1	0.75227027	0.03800335
P62761	Visinin-like protein 1	0.75188339	0.03511315
P47915	60S ribosomal protein L29	0.75064837	0.03818057
P60867	40S ribosomal protein S20	0.74677089	0.03788551
Q64487	Receptor-type tyrosine-protein phosphatase delta	0.74433298	0.04145827
Q8VDP6	CDP-diacylglycerol--inositol 3-phosphatidyltransferase	0.72462593	0.01199096
Q3UIU2	NADH dehydrogenase [ubiquinone] 1 beta subcomplex subunit 6	0.72405413	0.00536915
Q99KJ8	Dynactin subunit 2	0.71978629	0.01046852
Q9QYF9	Protein NDRG3	0.71952308	0.02883329
Q8R361	Rab11 family-interacting protein 5	0.71549771	0.03287298
Q91VZ6	Stromal membrane-associated protein 1	0.71496135	0.01691236
Q8K273	Membrane magnesium transporter 1	0.71270343	0.02652133
Q8VBT0	Thioredoxin-related transmembrane protein 1	0.69934635	0.02554594
Q6IRU5	Clathrin light chain B	0.68518463	0.04564995
O08677	Kininogen-1	0.67743259	0.03803371

Accession Number	Protein Name	ratio wt bgc vs. RKIP^{S153A} bgi	p value
P46061	Ran GTPase-activating protein 1	0.67123502	0.00341597
Q9QZI8	Serine incorporator 1	0.66756945	0.03847722
Q9Z2I2	Peptidyl-prolyl cis-trans isomerase FKBP1B	0.66220598	0.04901149
Q80ZS3	28S ribosomal protein S26, mitochondrial	0.65408903	0.02467241
Q8BYN5	FSD1-like protein	0.6483241	0.02003409
Q8K0E8	Fibrinogen beta chain	0.64278604	0.04423373
E9PV24	Fibrinogen alpha chain	0.62571547	0.02152053
Q9QUJ7	Long-chain-fatty-acid--CoA ligase 4	0.6241971	0.00656065
Q9JHL1	Na(+)/H(+) exchange regulatory cofactor NHE-RF2	0.61926181	0.04402647
Q9WTK3	Glycosylphosphatidylinositol anchor attachment 1 protein	0.61396645	0.01825288
Q9CS42	Ribose-phosphate pyrophosphokinase 2	0.61273627	0.00725406
Q3TXU5	Deoxyhypusine synthase	0.60652243	0.03918937
Q61207	Prosaposin	0.6033829	0.02562505
Q8BMI3	ADP-ribosylation factor-binding protein GGA3	0.59918578	0.01891376
P50428	Arylsulfatase A	0.58740193	0.03712415
O70503	Very-long-chain 3-oxoacyl-CoA reductase	0.5688275	0.04462659
Q64337	Sequestosome-1	0.56294753	0.01917978
Q00897	Alpha-1-antitrypsin 1-4	0.53533124	0.04682062
Q9D554	Splicing factor 3A subunit 3	0.52718264	0.00889563
P83887	Tubulin gamma-1 chain	0.52132379	0.00714183
P02468	Laminin subunit gamma-1	0.49556461	0.01536147
P23780	Beta-galactosidase	0.46642459	0.01599761
Q3UHG7	DENN domain-containing protein 11	0.45814364	0.03566528
Q68FD9	UPF0606 protein KIAA1549	0.45329904	0.04365417
Q8R0N6	Hydroxyacid-oxoacid transhydrogenase, mitochondrial	0.44377807	0.04308035
P22599	Alpha-1-antitrypsin 1-2	0.41808198	0.04557667
Q3UGX3	N-acetylaspartate synthetase	0.41479494	0.03406109
O09172	Glutamate--cysteine ligase regulatory subunit	0.40610974	0.01309108
P28665	Murinoglobulin-1	0.38077519	0.02600872
Q3UYG8	ADP-ribose glycohydrolase MACROD2	0.35805646	0.0224058
Q9CQH3	NADH dehydrogenase [ubiquinone] 1 beta subcomplex subunit 5, mitochondrial	0.24892982	0.02372628

Table S3

Significantly regulated proteins in wt bgc vs. RKIP^{S153A} bgi.

Mass spectrometric analyses of contralateral basal ganglia (bgc) of wild-type (wt; n = 4) mice and ipsilateral basal ganglia (bgi) of mice ubiquitously overexpressing a phosphorylation-

deficient mutant of RKIP^{S153A} (RKIP^{S153A}; n = 3) 24 h after transient middle cerebral artery occlusion. The table indicates the protein accession number, protein name, ratio and p-values; n-numbers represent samples of individual mice. For statistical analysis unpaired Students t-test was applied.

Table S4

Accession Number	Protein Name	ratio wt bgc vs. ERK2^{wt} bgi	p-value
Q3UHD1	Adhesion G protein-coupled receptor B1	9.8801142	0.00160508
Q80UP8	Sodium-dependent phosphate transporter 2	7.53376458	0.0150546
Q9D1J3	SAP domain-containing ribonucleoprotein	4.8011293	0.0004005
P20357	Microtubule-associated protein 2	4.00387032	0.02835547
Q68FM6	Protein phosphatase 1 regulatory subunit 29	3.92951167	0.03657407
Q9ERT9	Protein phosphatase 1 regulatory subunit 1A	3.73007877	0.00191003
Q9CZG9	PDZ domain-containing protein 11	3.55061552	4.5423E-05
P0C7L0	WAS/WASL-interacting protein family member 3	3.4391648	0.02104335
Q80X50	Ubiquitin-associated protein 2-like	3.42986684	0.00371725
Q922J3	CAP-Gly domain-containing linker protein 1	3.3897966	0.0040573
Q8C2Q3	RNA-binding protein 14	3.36855273	0.00770485
Q8CCJ4	APC membrane recruitment protein 2	3.25054147	0.00011253
P59644	Phosphatidylinositol 4,5-bisphosphate 5-phosphatase A	3.17024088	0.01652663
Q68ED7	CREB-regulated transcription coactivator 1	3.12801053	0.00250894
Q8VEA4	Mitochondrial intermembrane space import and assembly protein 40	3.07830327	0.02279605
Q810A7	ATP-dependent RNA helicase DDX42	3.06009109	0.02539841
Q9CY57	Chromatin target of PRMT1 protein	2.99161165	0.0134618
A2BDX3	Adenylyltransferase and sulfurtransferase MOCS3	2.82088655	0.00711041
P62309	Small nuclear ribonucleoprotein G	2.81898245	0.02498606
Q7TQH0	Ataxin-2-like protein	2.77461901	0.00122302
P97825	Jupiter microtubule associated homolog 1	2.75679285	0.01177425
O35684	Neuroserpin	2.75261993	0.04820619
Q8C0T5	Signal-induced proliferation-associated 1-like protein 1	2.73362913	0.00271452
Q8BG67	Protein EFR3 homolog A	2.71679639	0.0037232
Q80VC9	Calmodulin-regulated spectrin-associated protein 3	2.71311353	0.04095427
Q80WC7	Arf-GAP domain and FG repeat-containing protein 2	2.69299596	0.00457257
O88735	Ensconsin	2.64326146	0.00561821
P83510	Traf2 and NCK-interacting protein kinase	2.64235059	0.00084563
P62274	40S ribosomal protein S29	2.60684096	0.00708243
Q03173	Protein enabled homolog	2.55694557	0.03049297
Q7TSC1	Protein PRRC2A	2.5466745	0.00915536
Q9JM52	Misshapen-like kinase 1	2.46504957	0.00033875
Q58A65	C-Jun-amino-terminal kinase-interacting protein 4	2.41213187	0.01789932
Q8BG40	Katanin p80 WD40 repeat-containing subunit B1	2.39235368	0.00313321
Q71M36	Chondroitin sulfate proteoglycan 5	2.38737555	0.02231066

Accession Number	Protein Name	ratio wt bgc vs. ERK2^{wt} bgi	p-value
Q9WTS5	Teneurin-2	2.32182888	0.01648079
Q60598	Src substrate cortactin	2.32068822	0.02698646
Q9DC07	LIM zinc-binding domain-containing Nebulette	2.31301284	0.00619527
Q8CAK3	Shiftless antiviral inhibitor of ribosomal frameshifting protein homolog	2.29558942	0.03354717
Q8VI75	Importin-4	2.23729944	0.04165066
Q8BMS4	Ubiquinone biosynthesis O-methyltransferase, mitochondrial	2.21670379	0.01919515
B2RUJ5	Amyloid-beta A4 precursor protein-binding family A member 1	2.21621812	0.00879824
Q3UJB9	Enhancer of mRNA-decapping protein 4	2.21452525	0.00805639
O89086	RNA-binding protein 3	2.20080553	0.02608631
A2ALU4	Protein Shroom2	2.15474029	0.01641514
O88746	Target of Myb protein 1	2.14638503	0.01863721
P61957	Small ubiquitin-related modifier 2	2.13763049	0.04597012
Q9CQW0	ER membrane protein complex subunit 6	2.11943476	0.02242727
O70310	Glycylpeptide N-tetradecanoyltransferase 1	2.10981618	0.0093584
Q8C3Q5	Protein shisa-7	2.0943433	0.00657579
Q91W96	Anaphase-promoting complex subunit 4	2.09027453	0.03512145
Q99K01	Pyridoxal-dependent decarboxylase domain-containing protein 1	2.06587584	0.00958952
Q8VCN9	Tubulin-specific chaperone C	2.04067484	0.00515803
Q922V4	Pleiotropic regulator 1	2.0342809	0.00432566
Q9QXK3	Coatomer subunit gamma-2	2.02779807	0.01168987
Q8R317	Ubiquilin-1	2.02479529	0.04381852
F6SEU4	Ras/Rap GTPase-activating protein SynGAP	2.02286711	0.02997963
Q01097	Glutamate receptor ionotropic, NMDA 2B	2.01357297	0.03551263
Q80TE7	Leucine-rich repeat-containing protein 7	2.00968231	0.00587199
P60824	Cold-inducible RNA-binding protein	2.00413079	0.03612192
Q8R3Z5	Voltage-dependent L-type calcium channel subunit beta-1	1.98478691	0.01503851
Q8C0P5	Coronin-2A	1.98469898	0.0039485
P26040	Ezrin	1.97570713	0.01491031
Q6P9K8	Caskin-1	1.96897678	0.0047801
Q0VBD0	Integrin beta-8	1.9627976	0.0379429
P60670	Nuclear protein localization protein 4 homolog	1.9568934	0.0428499
P84086	Complexin-2	1.95512951	0.04455244
Q62417	Sorbin and SH3 domain-containing protein 1	1.95318853	0.00499957
O35927	Catenin delta-2	1.92412594	0.01101216
Q64378	Peptidyl-prolyl cis-trans isomerase FKBP5	1.92364937	0.026435
P10711	Transcription elongation factor A protein 1	1.9076494	0.00369299
Q9Z172	Small ubiquitin-related modifier 3	1.88703176	0.03222571
Q9Z130	Heterogeneous nuclear ribonucleoprotein D-like	1.87517742	0.02105121
P58802	TBC1 domain family member 10A	1.86731798	0.00264337

Accession Number	Protein Name	ratio wt bgc vs. ERK2^{wt} bgi	p-value
Q4VAA2	Protein CDV3	1.85876627	0.01638175
P62862	40S ribosomal protein S30	1.85795542	0.03611004
Q80TK0	AP2-interacting clathrin-endocytosis protein	1.85533355	0.02793101
Q8R1N4	NudC domain-containing protein 3	1.85282779	0.00100548
P60840	Alpha-endosulfine	1.83526737	0.00120212
Q8BL65	Actin-binding LIM protein 2	1.82884291	0.03728843
P97379	Ras GTPase-activating protein-binding protein 2	1.82586288	0.03533866
Q3UEB3	Poly(U)-binding-splicing factor PUF60	1.8202476	0.0092437
Q9CQN7	39S ribosomal protein L41, mitochondrial	1.82000787	0.01085264
Q6PD28	Serine/threonine-protein phosphatase 2A 56 kDa regulatory subunit beta isoform	1.81961442	0.01091803
P99027	60S acidic ribosomal protein P2	1.81396776	0.01281027
Q62448	Eukaryotic translation initiation factor 4 gamma 2	1.80772399	0.02637109
P63166	Small ubiquitin-related modifier 1	1.80560056	0.01802185
Q9DB10	Essential MCU regulator, mitochondrial	1.79877757	0.00328733
Q6R891	Neurabin-2	1.79808506	0.00761042
Q60936	Atypical kinase COQ8A, mitochondrial	1.79743562	0.00700785
Q9D2N4	Dystrobrevin alpha	1.79650561	0.03619811
Q9CQH7	Transcription factor BTF3 homolog 4	1.796199	0.01721064
P47964	60S ribosomal protein L36	1.78862767	0.0051478
Q91YQ3	Cold shock domain-containing protein C2	1.78403083	0.00331761
Q6PH08	ERC protein 2	1.73638311	0.00934805
Q91V36	Nuclear receptor-binding protein 2	1.72343367	0.02690623
Q9CR95	Adaptin ear-binding coat-associated protein 1	1.72291833	0.0016643
Q6A0A9	Constitutive coactivator of PPAR-gamma-like protein 1	1.72037312	0.03302154
O08579	Emerin	1.71951435	0.00018453
Q6P4T2	U5 small nuclear ribonucleoprotein 200 kDa helicase	1.71784241	0.01314348
Q9JKD3	Secretory carrier-associated membrane protein 5	1.71466457	0.0469743
Q9WV69	Dematin	1.71464431	0.00618575
Q0VBF8	Protein stum homolog	1.70881802	0.00711485
Q5SV85	Synergin gamma	1.6893194	0.02126378
Q8BHL3	TBC1 domain family member 10B	1.67680993	0.02316121
Q60902	Epidermal growth factor receptor substrate 15-like 1	1.67517779	0.04281649
Q4U4S6	Xin actin-binding repeat-containing protein 2	1.67448265	0.03135608
Q60714	Long-chain fatty acid transport protein 1	1.66674315	0.04000259
Q505D7	Optic atrophy 3 protein homolog	1.65670286	0.04447444
Q8VBY2	Calcium/calmodulin-dependent protein kinase kinase 1	1.65459781	0.00806933
Q8K010	5-oxoprolinase	1.6460311	0.02308182

Accession Number	Protein Name	ratio wt bgc vs. ERK2^{wt} bgi	p-value
Q8BH69	Selenide, water dikinase 1	1.6441216	0.00079009
Q9CY58	Plasminogen activator inhibitor 1 RNA-binding protein	1.63705067	0.01877676
P55096	ATP-binding cassette sub-family D member 3	1.63473844	0.02603675
O88737	Protein bassoon	1.6322399	0.01679651
Q8VDM6	Heterogeneous nuclear ribonucleoprotein U-like protein 1	1.62968879	0.02054641
Q3V3V9	Capping protein, Arp2/3 and myosin-I linker protein 2	1.62610069	0.01116651
Q60738	Zinc transporter 1	1.62029855	0.01437304
Q8BJI1	Sodium-dependent neutral amino acid transporter SLC6A17	1.6106434	0.04885429
Q64213	Splicing factor 1	1.60040459	0.01332544
Q9QWR8	Alpha-N-acetylgalactosaminidase	1.59803799	0.0142302
Q9Z0P4	Paralemmin-1	1.59505726	0.0468861
Q6PFE3	DNA repair and recombination protein RAD54B	1.5884517	0.02764672
Q5RJI5	Serine/threonine-protein kinase BRSK1	1.58827245	0.00102686
P18242	Cathepsin D	1.58354756	0.00772462
Q99K28	ADP-ribosylation factor GTPase-activating protein 2	1.57693462	3.3498E-05
D3YZI9	PiggyBac transposable element-derived protein 5	1.57347337	0.04393713
Q99J85	Neuronal pentraxin receptor	1.56864589	0.0433651
Q8CI71	Syndetin	1.56493819	0.03712425
Q9QX11	Cytohesin-1	1.56375788	0.04604059
Q3UHL1	CaM kinase-like vesicle-associated protein	1.56359934	0.01146297
Q99NE5	Regulating synaptic membrane exocytosis protein 1	1.5595898	0.00504326
Q99JY8	Phospholipid phosphatase 3	1.55564704	0.01966678
O89023	Tripeptidyl-peptidase 1	1.55205671	0.00283015
P61290	Proteasome activator complex subunit 3	1.54798554	0.0459117
O88745	Scrapie-responsive protein 1	1.54621482	0.00561391
P63040	Complexin-1	1.54579507	0.01865801
Q8K0G5	EARP and GARP complex-interacting protein 1	1.5333785	0.02047137
Q8VD33	Small glutamine-rich tetratricopeptide repeat-containing protein beta	1.53026897	0.04493799
Q8BGD9	Eukaryotic translation initiation factor 4B	1.52762371	0.04500451
P70206	Plexin-A1	1.52748302	0.00963497
Q62419	Endophilin-A2	1.52664673	0.04747814
Q8C1B1	Calmodulin-regulated spectrin-associated protein 2	1.52111676	0.04134556
Q9Z0H3	SWI/SNF-related matrix-associated actin-dependent regulator of chromatin subfamily B member 1	1.51874232	0.0078939
Q9D8W7	OCIA domain-containing protein 2	1.51858236	0.00136765

Accession Number	Protein Name	ratio wt bgc vs. ERK2^{wt} bgi	p-value
Q91W86	Vacuolar protein sorting-associated protein 11 homolog	1.51839854	0.00165026
P97797	Tyrosine-protein phosphatase non-receptor type substrate 1	1.51633455	0.01343782
Q8R071	Inositol-trisphosphate 3-kinase A	1.51593925	0.03142382
P56959	RNA-binding protein FUS	1.50923789	0.03798683
Q9JKC8	AP-3 complex subunit mu-1	1.50837848	0.01898126
Q3UHD9	Arf-GAP with GTPase, ANK repeat and PH domain-containing protein 2	1.49968809	0.00234181
Q08943	FACT complex subunit SSRP1	1.49889791	0.04818986
Q8VCE6	5'(3')-deoxyribonucleotidase, mitochondrial	1.49512327	0.00961184
Q9WUK2	Eukaryotic translation initiation factor 4H	1.49303429	0.00894656
Q9CQR2	40S ribosomal protein S21	1.49260906	0.04114491
P62317	Small nuclear ribonucleoprotein Sm D2	1.49042665	0.01446114
Q6A065	Centrosomal protein of 170 kDa	1.48946233	0.03594781
Q9ES28	Rho guanine nucleotide exchange factor 7	1.48605004	0.02174189
Q3UVX5	Metabotropic glutamate receptor 5	1.48421243	0.03506283
Q01063	cAMP-specific 3',5'-cyclic phosphodiesterase 4D	1.47675406	0.01209865
O88569	Heterogeneous nuclear ribonucleoproteins A2/B1	1.47470196	0.00285334
O88935	Synapsin-1	1.46668987	0.01927032
Q9CXZ1	NADH dehydrogenase [ubiquinone] iron-sulfur protein 4, mitochondrial	1.46417114	0.03717527
Q9EPJ9	ADP-ribosylation factor GTPase-activating protein 1	1.46324332	0.01534364
Q8C8N2	Protein SCAI	1.46304924	0.01236127
Q8VHK5	Membrane protein MLC1	1.46212837	0.02963591
Q9D7V2	LysM and putative peptidoglycan-binding domain-containing protein 2	1.45199414	0.02776094
Q99KN9	Clathrin interactor 1	1.45168422	0.02098347
Q03958	Prefoldin subunit 6	1.44958819	0.04187761
A6H5Z3	Exocyst complex component 6B	1.44229841	0.00030807
Q3UHJ0	AP2-associated protein kinase 1	1.44176103	0.04740192
P63325	40S ribosomal protein S10	1.44162611	0.01820806
P31230	Aminoacyl tRNA synthase complex-interacting multifunctional protein 1	1.43972564	0.04678002
Q9JK42	[Pyruvate dehydrogenase (acetyl-transferring)] kinase isozyme 2, mitochondrial	1.43810899	0.01660234
Q9QYR6	Microtubule-associated protein 1A	1.43505373	0.04024738
Q8K1E0	Syntaxin-5	1.43472922	0.04617594
Q99020	Heterogeneous nuclear ribonucleoprotein A/B	1.43264646	0.03893919
Q62418	Drebrin-like protein	1.43038684	0.02809732
Q8R0H9	ADP-ribosylation factor-binding protein GGA1	1.42868773	0.04783698
P70697	Uroporphyrinogen decarboxylase	1.42819009	0.04486021
Q9QWI6	SRC kinase signaling inhibitor 1	1.42523486	0.04486694

Accession Number	Protein Name	ratio wt bgc vs. $ERK2^{wt}$ bgi	p-value
Q62189	U1 small nuclear ribonucleoprotein A	1.42390081	0.03121942
Q8VD37	SH3-containing GRB2-like protein 3-interacting protein 1	1.42340302	0.01303703
Q9QYX7	Protein piccolo	1.42262036	0.00246459
Q9D0L8	mRNA cap guanine-N7 methyltransferase	1.42255399	0.04097004
Q91YP0	L-2-hydroxyglutarate dehydrogenase, mitochondrial	1.41554569	0.02752281
P97930	Thymidylate kinase	1.41459312	0.02246039
Q8BZJ7	DCN1-like protein 2	1.4140545	0.033343
Q8CHU3	Epsin-2	1.41117911	0.02140864
Q91XM9	Disks large homolog 2	1.40957136	0.0020581
P28028	Serine/threonine-protein kinase B-raf	1.40502324	0.01909551
P23198	Chromobox protein homolog 3	1.40434432	0.01659628
Q9ES00	Ubiquitin conjugation factor E4 B	1.40387271	0.04659524
Q91Z31	Polypyrimidine tract-binding protein 2	1.40325986	0.03280433
O70161	Phosphatidylinositol 4-phosphate 5-kinase type-1 gamma	1.4021823	0.01414528
P61979	Heterogeneous nuclear ribonucleoprotein K	1.39832878	0.03590384
Q91XD6	Vacuolar protein-sorting-associated protein 36	1.39747097	0.01480643
P54923	[Protein ADP-ribosylarginine] hydrolase	1.39569456	0.0257272
B9EJA2	Cortactin-binding protein 2	1.38922271	0.03185733
O35226	26S proteasome non-ATPase regulatory subunit 4	1.38742393	0.04182486
Q80U95	Ubiquitin-protein ligase E3C	1.38136459	0.04452554
Q9CZ44	NSFL1 cofactor p47	1.37839715	0.0044096
P53702	Cytochrome c-type heme lyase	1.3770181	0.00162252
Q3UHX2	28 kDa heat- and acid-stable phosphoprotein	1.37658738	0.02777982
Q9QYC0	Alpha-adducin	1.36946418	0.00750434
Q91V09	WD repeat-containing protein 13	1.36893503	0.02479254
Q8CDG3	Deubiquitinating protein VCIP135	1.36669667	0.00599869
Q9JII5	DAZ-associated protein 1	1.36492707	0.00406415
P47809	Dual specificity mitogen-activated protein kinase kinase 4	1.36223932	0.02973226
P70288	Histone deacetylase 2	1.36078499	0.01081087
Q80TV8	CLIP-associating protein 1	1.36047584	0.02992935
Q9Z0R4	Intersectin-1	1.3603762	0.01109147
P55066	Neurocan core protein	1.35417089	0.01902548
P62313	U6 snRNA-associated Sm-like protein LSm6	1.34916413	0.03374413
O88845	A-kinase anchor protein 10, mitochondrial	1.34754697	0.01220228
Q8K1N1	Calcium-independent phospholipase A2-gamma	1.34641721	0.00207136
P35288	Ras-related protein Rab-23	1.34499576	0.02489696
Q91W90	Thioredoxin domain-containing protein 5	1.33987326	0.04427314
Q8C8T8	Pre-rRNA-processing protein TSR2 homolog	1.33875787	0.0403834
Q9CQJ6	Density-regulated protein	1.33444072	0.00126635

Accession Number	Protein Name	ratio wt bgc vs. ERK2^{wt} bgi	p-value
Q8K310	Matrin-3	1.32919496	0.01879213
O88441	Metaxin-2	1.32853115	0.00040433
Q8BRT1	CLIP-associating protein 2	1.32432037	0.04543549
Q9WUM3	Coronin-1B	1.32238548	0.04713588
Q9DC28	Casein kinase I isoform delta	1.31931325	0.04763282
O35551	Rab GTPase-binding effector protein 1	1.31876942	0.00235073
Q9Z2H5	Band 4.1-like protein 1	1.31592651	0.04155215
Q80TZ3	Putative tyrosine-protein phosphatase auxilin	1.31272685	0.02387171
P54823	Probable ATP-dependent RNA helicase DDX6	1.30506465	0.03943496
Q8CGF7	Transcription elongation regulator 1	1.30011078	0.0409104
O70318	Band 4.1-like protein 2	1.29363	0.0450851
P14869	60S acidic ribosomal protein P0	1.29248976	0.04077861
O70493	Sorting nexin-12	1.29133051	0.01740459
P51859	Hepatoma-derived growth factor	1.29114403	0.0325582
Q8C052	Microtubule-associated protein 1S	1.28810504	0.03256014
Q8BNY6	Neuronal calcium sensor 1	1.28596298	0.04113088
P60469	Liprin-alpha-3	1.28478905	0.01728787
Q8BMF4	Dihydrolipoyllysine-residue acetyltransferase component of pyruvate dehydrogenase complex, mitochondrial	1.28241347	0.04261364
P28652	Calcium/calmodulin-dependent protein kinase type II subunit beta	1.2808999	0.03905616
Q80U63	Mitofusin-2	1.27881674	0.03374923
Q8R5H6	Wiskott-Aldrich syndrome protein family member 1	1.27746389	0.01930468
Q9CRD0	OCIA domain-containing protein 1	1.27453917	0.02338003
Q7TN29	Stromal membrane-associated protein 2	1.27324329	0.01741506
Q8CGY8	UDP-N-acetylglucosamine--peptide N-acetylglucosaminyltransferase 110 kDa subunit	1.27269436	0.03623602
Q61656	Probable ATP-dependent RNA helicase DDX5	1.27215081	0.01380146
Q60996	Serine/threonine-protein phosphatase 2A 56 kDa regulatory subunit gamma isoform	1.270946	0.02928831
O55042	Alpha-synuclein	1.2706332	0.03750141
Q8R2R9	AP-3 complex subunit mu-2	1.26865613	0.04431507
Q8BUV3	Gephyrin	1.26607915	0.02602459
P51910	Apolipoprotein D	1.26401294	0.01842865
Q6PIP5	NudC domain-containing protein 1	1.26383622	0.0239394
P48774	Glutathione S-transferase Mu 5	1.26341828	0.02442345
Q6PFE7	Tomoregulin-1	1.26245151	0.03983451
O08709	Peroxiredoxin-6	1.26213053	0.01094014
Q8R3S6	Exocyst complex component 1	1.24221782	0.03605254
Q6TL19	Guanylate cyclase 2G	1.23599166	0.04490222
Q9R0H0	Peroxisomal acyl-coenzyme A oxidase 1	1.23588441	0.00740768
Q9EPN1	Neurobeachin	1.23486905	0.04220444
Q9QYB8	Beta-adducin	1.22850534	0.00637534

Accession Number	Protein Name	ratio wt bgc vs. ERK2^{wt} bgi	p-value
P63011	Ras-related protein Rab-3A	1.22357275	0.03399316
O35382	Exocyst complex component 4	1.22194988	0.04521325
P21550	Beta-enolase	1.21987238	0.04121552
P57746	V-type proton ATPase subunit D	1.21716732	0.00496507
Q9CPU0	Lactoylglutathione lyase	1.21025582	0.01649526
Q9JHR7	Insulin-degrading enzyme	1.20978573	0.01576164
P22315	Ferrochelatase, mitochondrial	1.20969319	0.03487524
O35621	Phosphomannomutase 1	1.20853247	0.01224336
Q9D051	Pyruvate dehydrogenase E1 component subunit beta, mitochondrial	1.2041551	0.00031555
Q3V3R1	Monofunctional C1-tetrahydrofolate synthase, mitochondrial	1.19893964	0.0351666
P70296	Phosphatidylethanolamine-binding protein 1	1.19818818	0.00182446
Q60631	Growth factor receptor-bound protein 2	1.19712469	0.02416349
O35643	AP-1 complex subunit beta-1	1.19602102	0.03477981
P62852	40S ribosomal protein S25	1.19529371	0.03416339
P61087	Ubiquitin-conjugating enzyme E2 K	1.19477535	0.0245264
Q8R326	Paraspeckle component 1	1.18843843	0.00920927
Q91ZJ5	UTP--glucose-1-phosphate uridylyltransferase	1.1782271	0.0284479
Q2NL51	Glycogen synthase kinase-3 alpha	1.17440759	0.03254077
Q9JI46	Diphosphoinositol polyphosphate phosphohydrolase 1	1.17075861	0.00142837
P17182	Alpha-enolase	1.16917124	0.04990118
Q9CZY3	Ubiquitin-conjugating enzyme E2 variant 1	1.16616668	0.01678182
Q8BVE3	V-type proton ATPase subunit H	1.1628437	0.04125889
P62141	Serine/threonine-protein phosphatase PP1-beta catalytic subunit	1.15303832	0.00578379
Q922H2	[Pyruvate dehydrogenase (acetyl-transferring)] kinase isozyme 3, mitochondrial	1.1425482	0.01525136
P48962	ADP/ATP translocase 1	1.13729207	0.04863647
Q8BLF1	Neutral cholesterol ester hydrolase 1	1.13687016	0.02680499
P60710	Actin, cytoplasmic 1	1.13534	0.01116305
P17742	Peptidyl-prolyl cis-trans isomerase A	1.13113979	0.02444844
Q5HZI9	Solute carrier family 25 member 51	1.12788581	0.00520508
Q9R257	Heme-binding protein 1	1.12632619	0.02516023
P22892	AP-1 complex subunit gamma-1	1.10622365	0.014942
P16858	Glyceraldehyde-3-phosphate dehydrogenase	1.07837371	0.04215505
P63001	Ras-related C3 botulinum toxin substrate 1	0.90620516	0.03156982
Q56A07	Sodium channel subunit beta-2	0.88659172	0.03833206
Q3TZZ7	Extended synaptotagmin-2	0.88028657	0.0444236
Q9JHU4	Cytoplasmic dynein 1 heavy chain 1	0.86284217	0.01261206
Q9WV55	Vesicle-associated membrane protein-associated protein A	0.8610504	0.02978149
Q8BH66	Atlastin-1	0.85352141	0.03844798
Q8BYR1	tRNA wybutosine-synthesizing protein 4	0.83295469	0.0396347

Accession Number	Protein Name	ratio wt bgc vs. ERK2^{wt} bgi	p-value
P09528	Ferritin heavy chain	0.82593522	0.02904298
Q9D1L9	Ragulator complex protein LAMTOR5	0.82515953	0.0433378
Q9JMC3	DnaJ homolog subfamily A member 4	0.81245258	0.04663475
Q9JIW9	Ras-related protein Ral-B	0.80422301	0.04384108
Q8CHH9	Septin-8	0.80353054	0.01424376
Q9WV85	Nucleoside diphosphate kinase 3	0.80032058	0.0341844
Q9QZB9	Dynactin subunit 5	0.79185488	0.00614483
Q61335	B-cell receptor-associated protein 31	0.78071866	0.02528127
P57716	Nicastrin	0.77561623	0.01988276
Q7TPM6	Fibronectin type III and SPRY domain-containing protein 1	0.77341033	0.02723873
Q60605	Myosin light polypeptide 6	0.7704992	0.01038908
Q9QYI5	DnaJ homolog subfamily B member 2	0.76326524	0.03384039
Q9D517	1-acyl-sn-glycerol-3-phosphate acyltransferase gamma	0.76165806	0.01556884
P19536	Cytochrome c oxidase subunit 5B, mitochondrial	0.75682202	0.03051166
Q63912	Oligodendrocyte-myelin glycoprotein	0.75579122	0.01888242
Q8VEH3	ADP-ribosylation factor-like protein 8A	0.75146742	0.01031292
A2ASZ8	Calcium-binding mitochondrial carrier protein SCaMC-2	0.74522771	0.03459163
P97370	Sodium/potassium-transporting ATPase subunit beta-3	0.7445243	0.02091303
Q99P31	Hsp70-binding protein 1	0.73710289	0.02348929
P12787	Cytochrome c oxidase subunit 5A, mitochondrial	0.7343861	0.025389
P35279	Ras-related protein Rab-6A	0.7254427	0.00578894
Q8R366	Immunoglobulin superfamily member 8	0.71792485	0.01872633
Q9QYB1	Chloride intracellular channel protein 4	0.71711199	0.04782563
Q8VD75	Huntingtin-interacting protein 1	0.70957406	0.03351572
Q8VBT0	Thioredoxin-related transmembrane protein 1	0.70251655	0.0237628
O70172	Phosphatidylinositol 5-phosphate 4-kinase type-2 alpha	0.70194197	0.04564927
Q9D3P8	Plasminogen receptor (KT)	0.69361813	0.04395911
Q9EP69	Phosphatidylinositide phosphatase SAC1	0.68831101	0.00245858
O70503	Very-long-chain 3-oxoacyl-CoA reductase	0.68577074	0.03351747
Q61553	Fascin	0.67413086	0.04075929
Q8BYN5	FSD1-like protein	0.67323487	0.02604262
Q9QY23	Plakophilin-3	0.67182156	0.01170997
Q8BMI3	ADP-ribosylation factor-binding protein GGA3	0.66326662	0.03604343
P62838	Ubiquitin-conjugating enzyme E2 D2	0.65304754	0.02783634
P00920	Carbonic anhydrase 2	0.64400466	0.04467942
P21460	Cystatin-C	0.64146054	0.03517855
P01887	Beta-2-microglobulin	0.6391905	0.00293038
Q8K4Z0	Leucine-rich repeat LGI family member 2	0.63452157	0.03282051

Accession Number	Protein Name	ratio wt bgc vs. ERK2^{wt} bgi	p-value
Q61712	DnaJ homolog subfamily C member 1	0.63373615	0.00616552
P23927	Alpha-crystallin B chain	0.62975085	0.00487626
Q5BJ29	F-box/LRR-repeat protein 7	0.62558228	0.03880054
P83887	Tubulin gamma-1 chain	0.6038292	0.0482227
P28661	Septin-4	0.60365002	0.02285726
Q06335	Amyloid-like protein 2	0.60052405	0.01355713
O08807	Peroxiredoxin-4	0.59666199	0.00525331
P13020	Gelsolin	0.59141661	0.04238176
Q8BH82	N-acyl-phosphatidylethanolamine-hydrolyzing phospholipase D	0.57864273	0.0378619
Q5SVL6	Rap1 GTPase-activating protein 2	0.56954269	0.02742968
Q9JI19	Acidic fibroblast growth factor intracellular-binding protein	0.56810972	0.00427562
P16283	Anion exchange protein 3	0.56402044	0.04115812
Q8BZA9	Fructose-2,6-bisphosphatase TIGAR	0.56393651	0.03883182
P19246	Neurofilament heavy polypeptide	0.55238083	0.03992242
P08553	Neurofilament medium polypeptide	0.54665978	0.04925923
P16330	2',3'-cyclic-nucleotide 3'-phosphodiesterase	0.5400812	0.04257794
P40237	CD82 antigen	0.52615166	0.0279098
P12023	Amyloid-beta A4 protein	0.52436881	0.00542023
Q9EPL2	Calsyntenin-1	0.52281589	0.01870699
Q9QZI8	Serine incorporator 1	0.51775088	0.00874765
Q02013	Aquaporin-1	0.44484731	0.02875802
Q9WVT6	Carbonic anhydrase 14	0.42369923	0.00231103
Q8R4V2	Dual specificity protein phosphatase 15	0.37374363	0.02410295
O09172	Glutamate--cysteine ligase regulatory subunit	0.36245072	0.0028391
P56375	Acylphosphatase-2	0.30793137	0.00018954
Q99JH7	Calsyntenin-3	0.30514815	0.04520415
Q3UHG7	DENN domain-containing protein 11	0.26016027	0.02163239
Q9DBB8	Trans-1,2-dihydrobenzene-1,2-diol dehydrogenase	0.24900268	0.02385696
P60954	Nucleolar protein 4	0.18849156	0.02637624

Table S4

Significantly regulated proteins in wt bgc vs. ERK2^{wt} bgi.

Mass spectrometric analyses of contralateral basal ganglia (bgc) of wild-type (wt) mice and ipsilateral basal ganglia (bgi) of mice ubiquitously overexpressing ERK2^{wt} (ERK2^{wt}) 24 h after transient middle cerebral artery occlusion (n = 4). The table indicates the protein accession

number, protein name, ratio and p-values; n-numbers represent samples of individual mice. For statistical analysis unpaired Students t-test was applied.

Table S5

Accession Number	Protein Name	bgi wt 1	bgi wt 2	bgi wt 3	bgi wt 4	bgi wt 5	bgi $ERK2^{wt}$ 1	bgi $ERK2^{wt}$ 2	bgi $ERK2^{wt}$ 3	bgi $ERK2^{wt}$ 4	bgi $RKIP^{wt}$ 1	bgi $RKIP^{wt}$ 2	bgi $RKIP^{S153A}$ 1	bgi $RKIP^{S153A}$ 2	bgi $RKIP^{S153A}$ 3
P16330	2',3'-cyclic-nucleotide 3'-phosphodiesterase	3453037437	3211812266	2565931310	2317250363	1448022575	5102171347	3959862486	2940395988	4382283677	4388319989	2025024638	2281908767	2166746739	2621916949
Q04447	Creatine kinase B-type	1541702178	2044552818	1683160943	2267145928	1430711290	2587528859	2999089493	2010170656	2324487223	1511773188	2169458435	2189545630	1808050232	1411068256
P14094	Sodium/potassium-transporting ATPase subunit beta-1	2213973996	1928624603	2091388378	2092832801	2456576620	1841238504	1888234556	2065872444	1790739332	2366101586	2197288173	2099140832	2301338514	2266872837
P08553	Neurofilament medium polypeptide	896554946	1130476577	760679707	916695245	638841777	1602021149	1737382471	1038986849	1593505420	1313240302	1052941628	864056470	884000041	878350187
P46660	Alpha-internexin	837866109	914662448	495824402	619932205	483304640	1167721391	1253894801	728666592	1173077164	854447226	622221153	762092079	577308915	589781126
P08551	Neurofilament light polypeptide	771086527	796928399	574062113	573538893	402385053	1119542085	1201996323	674248279	1117777397	972956127	605257962	660684327	583168835	588294820
P17710	Hexokinase-1	1133023415	1025259931	1219542759	1083200064	1094830967	859508931	1012133185	1024830988	989582787	1037948767	1037610427	1154217978	1036491153	1111451836
P62814	V-type proton ATPase subunit B, brain isoform	746471309	849309074	864210509	762595030	759097473	628550041	707772258	732227104	723088056	715617068	744202246	864195941	761841921	832050274
P68369	Tubulin alpha-1A chain	468609046	538076736	557722593	444913430	496615663	643997314	523644161	617787747	676606416	578333740	339858546	493184984	521080109	464149854
P62897	Cytochrome c, somatic	457841654	416639170	470751768	507164693	459835029	482742047	523176558	514554620	526699781	574171516	562424781	419490744	588045806	538894600
Q64332	Synapsin-2	700882225	604818869	700812807	531661385	641976302	372886280	444372202	563812704	524627701	539826081	448992378	590380592	561572527	681424190
P62983	Ubiquitin-40S ribosomal protein S27a	346672613	429422855	371442272	383877975	302768679	376498983	467553509	442181863	470431542	556254143	522071106	379950075	422739542	427454958
Q8QZT1	Acetyl-CoA acetyltransferase, mitochondrial	363327672	415566886	389122789	444080044	447534782	360001757	368818941	389388464	345106128	393821262	441334113	310579756	391420021	427495995
P19246	Neurofilament heavy polypeptide	209622365	279915887	201762494	190762151	183714995	407290924	367428292	224596239	354623744	459851702	164991198	207021657	220370102	218028822

Accession Number	Protein Name	bgi wt 1	bgi wt 2	bgi wt 3	bgi wt 4	bgi wt 5	bgi ERK2^{wt} 1	bgi ERK2^{wt} 2	bgi ERK2^{wt} 3	bgi ERK2^{wt} 4	bgi RKIP^{wt} 1	bgi RKIP^{wt} 2	bgi RKIP^{S153A} 1	bgi RKIP^{S153A} 2	bgi RKIP^{S153A} 3
Q91VD9	NADH-ubiquinone oxidoreductase 75 kDa subunit, mitochondrial	327661887	311582642	287896639	324266265	350401966	277572881	303365722	302477136	275343017	308583769	331556268	299477961	321726157	295395754
Q9D051	Pyruvate dehydrogenase E1 component subunit beta, mitochondrial	279648706	250115144	283799238	248153760	283270667	242668263	220763648	238198443	237264232	268095042	250144615	275614370	250579590	257752596
Q8BMF4	Dihydrolipoyllysine-residue acetyltransferase component of pyruvate dehydrogenase complex, mitochondrial	280194826	242971994	250260717	213299394	295650396	198010552	223700673	219646778	199682108	272777756	234845020	253897865	260375457	272085496
Q9DCX2	ATP synthase subunit d, mitochondrial	196731208	266576657	314013074	258696240	323987218	178978669	206238750	193105016	221426990	273724601	283737636	200310116	256824553	297240678
P12787	Cytochrome c oxidase subunit 5A, mitochondrial	143571160	140740555	132266915	151968111	151067848	171491728	203464340	166554725	162947053	113701891	150759405	138323663	152184364	159765643
P63044	Vesicle-associated membrane protein 2	228103615	312035281	256245834	233716234	176031417	131129467	185810254	197397033	184371653	151158120	231526115	284834973	219292331	196652133
Q8BLK3	Limbic system-associated membrane protein	146160179	184645435	137435576	148613895	164104791	119079765	115264635	127204481	137582785	113880320	143575380	134138649	126863286	142548025
Q9CPQ8	ATP synthase subunit g, mitochondrial	144113950	134339447	150472136	124479178	156919040	105836416	129560954	104253641	125915639	120138386	151177988	115827085	138580308	142642514
Q9CQA3	Succinate dehydrogenase [ubiquinone] iron-sulfur subunit, mitochondrial	96933529.8	117069446	113687920	124127650	127015110	91404537.7	97440948.1	104444667	107809357	126045340	149151484	102116009	124523055	126446491

Accession Number	Protein Name	bgi wt 1	bgi wt 2	bgi wt 3	bgi wt 4	bgi wt 5	bgi ERK2^{wt} 1	bgi ERK2^{wt} 2	bgi ERK2^{wt} 3	bgi ERK2^{wt} 4	bgi RKIP^{wt} 1	bgi RKIP^{wt} 2	bgi RKIP^{S153A} 1	bgi RKIP^{S153A} 2	bgi RKIP^{S153A} 3
Q80YN3	Breast carcinoma-amplified sequence 1 homolog	91261416.1	79936604.2	68596471.5	61772796.6	29321215.4	82596136.7	111102838	90995397	113769352	100129823	69425367.5	59425424.8	74909135.8	74968670.1
Q9D2P8	Myelin-associated oligodendrocyte basic protein	78669935.6	40209954.1	40074959.3	40707600.5	23314621.6	93850651.9	77884185.9	53773254.2	76195461.9	75347110.2	33525885.7	44614564	37817181.7	45688399.1
Q9D6J6	NADH dehydrogenase [ubiquinone] flavoprotein 2, mitochondrial	79983258.6	80377442.4	78185125.7	69901175.3	85357614	70757407.8	65366647.4	70422259.4	73224565.6	71899864.5	82819071.2	82319296.2	74896178.5	79814919
G5E8K5	Ankyrin-3	49403826.6	49825848.4	44846457.7	46071609.7	34941904.6	59525504.1	56186181.1	48052167.9	54638626.7	45874139.6	49912602.8	63944864.9	43606581.4	44161071.4
Q99JR1	Sideroflexin-1	77929087.4	60165410.9	56507924.7	60786256.9	54641339.5	43151673	45742588.3	55164416.6	50738232.1	50523387.3	61515877.2	53164327.8	70125020.9	58675487.1
Q9CPU0	Lactoylglutathione lyase	62158290.4	55027212.1	64105999.6	45321566.2	72272470.4	52757233.3	43936312.8	43139247.2	46960551.4	55905279.1	45083262	46127475.8	43528209.3	58308415.1
Q9R1V7	Disintegrin and metalloproteinase domain-containing protein 23	30958032.4	31925591.5	29785422.1	34458264.3	22624039.1	47864995.4	42643651.6	38141473	32326505.9	40159854.7	34296766.2	37203507.2	30978825.1	26359315.9
Q62418	Drebrin-like protein	35702333.6	36423197.5	41107460.7	44047108.5	45925840	27629317.3	34162940.7	36833225.1	35944247.8	43442985.1	43566905.8	43018704	41169087.8	45010412.2
Q8R5M8	Cell adhesion molecule 1	46717233.1	40357639.4	33074135	35094851.1	48121335.1	25223635.9	21078101.8	36054294.2	30496698.3	30922559.9	35649199.4	36642157.2	43184731.8	39217470.2
Q9QXZ0	Microtubule-actin cross-linking factor 1	31906533.8	36052767.3	34871075.3	30515272.4	44200609.1	21453973.1	27309305.4	29903886.8	31678107.4	28639070.4	31283544.8	34887230.4	37906156.9	37741249.8
P28661	Septin-4	24769612	17057712.2	20431215.9	14410731.6	15128130.5	28287396.7	31860312.7	23487690.3	23328673.4	29029366.7	14145382.2	25608708	14402697.4	24056233
Q80U28	MAP kinase-activating death domain protein	26155107.8	33654449.5	30922796	31754788.8	33551967.2	27508069.6	26927705.2	25051065	26943721.1	22627580.3	31761441.3	36027145.4	26366630.7	36024737
O08917	Flotillin-1	43388790.4	36465697.3	38907595	28646768.3	30964327	23345317.2	22260072	24754120.9	30992099.3	29853552.3	24198858.8	32956310.1	27968221.4	37214403.1

Accession Number	Protein Name	bgi wt 1	bgi wt 2	bgi wt 3	bgi wt 4	bgi wt 5	bgi ERK2^{wt} 1	bgi ERK2^{wt} 2	bgi ERK2^{wt} 3	bgi ERK2^{wt} 4	bgi RKIP^{wt} 1	bgi RKIP^{wt} 2	bgi RKIP^{S153A} 1	bgi RKIP^{S153A} 2	bgi RKIP^{S153A} 3
Q60634	Flotillin-2	39453849.9	31443237.1	39924771	24457558.8	40578078.5	19633021.7	19890870.1	27488914	25147197.4	33160316.3	26818577.9	31143316.7	28165557.7	35325222
Q8C078	Calcium/calmodulin-dependent protein kinase kinase 2	3349576.73	18976058.3	9603430.23	22551543.3	9871888.67	21658186.4	25922667.2	18569549.1	24570219.8	7102796.66	28698757.8	26772932.3	15819209.3	12843194
Q71LX4	Talin-2	31105965.1	27015945.6	27484113	25586885.7	29390483.2	20664769.6	18778457.4	27313100.8	21770032.5	22216260.1	25014795.4	22911374.9	24616963.9	25572298.3
Q9WV80	Sorting nexin-1	31147697.6	22075038.5	29148745.6	27009410.9	23529642.6	20367224.4	24326342	20967077.9	21466595.1	23441226	17691078.4	20598996.3	21123732.8	19946905.6
P34022	Ran-specific GTPase-activating protein	26880386.6	27901429.8	21665330.7	25642863.1	25951314	19479969.8	21445810.5	24833814.5	21269953.2	26810529.6	26766991.2	20609286.6	28611868.2	27874271.9
Q9D8B4	NADH dehydrogenase [ubiquinone] 1 alpha subcomplex subunit 11	21511066.2	22995396.6	26075463.3	23581391.1	21781973.2	21346628.5	20560283.3	20766002.3	20358612.7	25302126.7	25277494.1	25021651.3	19805283.8	19976395.4
O70172	Phosphatidylinositol 5-phosphate 4-kinase type-2 alpha	9502329.52	14968261	11258142.6	17261206.1	10733789.6	19410502.4	20149584.3	17847804.1	21472155.7	16826961	16149622.6	18587873.2	14295350.3	13230779.7
Q8R016	Bleomycin hydrolase	13074851.7	10398429.5	15380447.2	10373961.6	13492244.4	22943385	20667630	17660299.2	15847632.5	16625166.6	13129515.4	12539307.2	12144718.5	14439388.7
O88545	COP9 signalosome complex subunit 6	22072541.8	21037023.6	23491181.6	20204291	27463961.5	17804686.4	17773648.4	19837521.7	19240104.3	20080318	17049773.5	14802825.6	17336593.7	21221216.5
Q99JY8	Phospholipid phosphatase 3	29999421.9	19185501.1	29122506.5	19716158	24661905.6	17099229	13516103.3	21321041.5	18450884.9	20325177.8	18134913.3	20767947.6	20953046.3	21557153.6
Q9Z0Y1	Dynactin subunit 3	22305043.6	19202000.8	18953825	19952223.6	21690603.7	15058983.1	17925983.9	17880249.9	18853958.5	18577242.8	19779072.2	17737493.7	23101753.1	21632476.3
P16332	Methylmalonyl-CoA mutase, mitochondrial	18050089.2	19823333.6	21431593.3	20332111.3	19589895.7	18170233.5	16888660.8	17401177.6	16484249	13825895	18267617.6	23473041.8	15653313.2	13629483.1
P43024	Cytochrome c oxidase subunit 6A1, mitochondrial	7250735.09	9953275.77	10131581.4	6818761.46	9666785.42	14182485.6	21769822.5	10700620	17344745.1	6070108.26	6767053.72	7187750.22	11763954.1	11958316.9

Accession Number	Protein Name	bgi wt 1	bgi wt 2	bgi wt 3	bgi wt 4	bgi wt 5	bgi ERK2^{wt} 1	bgi ERK2^{wt} 2	bgi ERK2^{wt} 3	bgi ERK2^{wt} 4	bgi RKIP^{wt} 1	bgi RKIP^{wt} 2	bgi RKIP^{S153A} 1	bgi RKIP^{S153A} 2	bgi RKIP^{S153A} 3
Q9D7N9	Adipocyte plasma membrane-associated protein	19378927.7	17279156.1	16156030.2	18732666	18316104.9	14776851.7	15982154	17420934	14962964	16895115.1	16952924.6	15519311.6	19309708.1	17126188
Q9ER00	Syntaxin-12	20168564.6	18921036.6	17527667.4	19396239.2	18999389	12768399.2	14983553	18265121.5	15428439.4	18448486.8	17044431.2	16558419	18871902.8	18369485.1
Q9Z268	RasGAP-activating-like protein 1	20051828.2	23916561	24872123.3	16342771.4	33166596.5	13633192.4	14176075.6	14741847.7	18728500.8	17327159.1	22589148.4	27449850.9	17192286	30000890
Q9EPW0	Inositol polyphosphate-4-phosphatase type I A	12782123.3	13460928.6	10861048.2	11900884.5	8841208.6	14149777.3	14298183.7	13506634.7	15955050.1	11497121.8	13414838	13561670.6	11525429.3	11409095.3
Q3V3R1	Monofunctional C1-tetrahydrofolate synthase, mitochondrial	17784861.3	15370558.6	14665735.6	17413530.6	17105311.6	15016442.6	13181288.2	14731855.8	13469259.2	16670763.1	17079274.6	15534095.1	13159036.1	14971882.5
Q99PL6	UBX domain-containing protein 6	10743165	12207241.8	12567427.2	14157015.4	10466400.5	12860659.9	14679878.7	13635519.1	14783159.7	11915619.5	11419417.5	14772217.2	13659808.9	13269079.6
Q3UHD6	Sorting nexin-27	18250014.9	15973481.7	18633025.9	16650136.1	13879483.2	14785874.7	12047136.3	14120830.5	14214608.5	12921161.7	12114672.9	15071634	13798566.4	15486001.9
Q9CRD0	OCIA domain-containing protein 1	13499005	13745610.6	12648728	16045494.9	15016049.1	10085099.5	11699854.9	12611186.5	12121382.6	13655768.7	14493589.4	11875143.8	15112597.7	15666385.5
Q3UUG6	TBC1 domain family member 24	15799276.2	14204607.1	19512699.9	14659476.8	15079702	10374959	11109401.5	13396932.2	11595207.6	15198243.4	15034887.4	13836469.6	14107107.1	16916143.1
Q9WVK4	EH domain-containing protein 1	14310560.1	13009186.7	16740753.6	11743267.8	14202933.6	9115657.79	10865761.1	11336235.4	9842132.48	11145761.9	9460167.66	11207355.6	10596568.3	17582878.5
Q99LB6	Methionine adenosyltransferase 2 subunit beta	6219176.58	8390835.69	7512771.35	8532652.64	6553667.09	11548932.7	10006747.9	8218740.09	9311439.94	6160009.34	9517304.38	8727623.07	7915264.62	7091582.51
P57776	Elongation factor 1-delta	15283173.5	15153912.1	16472039.6	9645691.76	20355187	10044147.7	9442021.61	10504711.7	8378202.23	14882785.2	9552317.61	17539950.2	15284555.8	10943677.8
Q8BNY6	Neuronal calcium sensor 1	11220066.6	11477023	12304498.7	12641252.5	15559270.3	9241759.48	8745228.39	9873252.19	10242534.9	8423378.69	9409632.83	8803331.24	8205812.17	12096006.1
Q6P8X1	Sorting nexin-6	11866350.8	10350095.2	13973865.3	8982554.27	12224456.4	9021949.53	9474007.63	7913536.69	7087497.78	7457330.98	6852278.55	8170508.92	10170655.1	10955150.2
P56375	Acylphosphatase-2	2926906.76	7668903.6	4558924.33	2376462.33	4269969.05	9224503.89	8120694.47	7585216.94	7077693.94	6315040.52	5960134.06	3676929	5499268.51	2636472.5

Accession Number	Protein Name	bgi wt 1	bgi wt 2	bgi wt 3	bgi wt 4	bgi wt 5	bgi $ERK2^{wt}$ 1	bgi $ERK2^{wt}$ 2	bgi $ERK2^{wt}$ 3	bgi $ERK2^{wt}$ 4	bgi $RKIP^{wt}$ 1	bgi $RKIP^{wt}$ 2	bgi $RKIP^{S153A}$ 1	bgi $RKIP^{S153A}$ 2	bgi $RKIP^{S153A}$ 3
Q9ESJ4	NCK-interacting protein with SH3 domain	10551289.9	8915042.26	13657571.2	9211864.02	11262958.2	6568251.17	7000936.04	9260580.09	8509384.84	12379373.4	9381062.34	13383016.8	10507507.7	14477929.7
Q8BMJ2	Leucine--tRNA ligase, cytoplasmic	10940252.7	9088515.76	10277226.8	10739385.9	10376814.8	7870365.16	4981092.27	8764875.42	6988317.34	9274989.17	10629464.9	11238093	11393173.2	10455004.2
Q80X95	Ras-related GTP-binding protein A	4017213.07	5548710.93	5789364.87	5121077.04	4348518.96	6019169.08	7455898.17	5850924.69	7336229.97	6181720.99	8304587.64	4115098.58	4183278.84	3482165.8
P70206	Plexin-A1	10481309.2	8916801.71	8624203.85	6659444.16	10597939.2	6173655.65	6074539.88	7353726.65	6727074.35	7132109.05	8590556.68	7364857.68	5195308.84	9279047.92
Q8BZA9	Fructose-2,6-bisphosphatase TIGAR	4156868.98	3367783.84	5154102.51	4953119.41	1716498.68	6678314.36	5056412.25	6047497.18	6799127.48	3182771.81	4245047.71	4624478.37	3203939.21	2697311.32
Q8C0L0	Thioredoxin-related transmembrane protein 4	9368343.03	6813612.52	9001930.18	6408717.02	6890059.52	4909396.58	6328655.87	6541580.26	4827704.85	5750304.37	6617952.78	10814953.9	7592424.7	4164116.62
P60764	Ras-related C3 botulinum toxin substrate 3	7017471.95	6255684.47	6626056.49	7693382.55	5880318.44	4411357.28	5304791.17	6115255.33	5530338.77	6013698.6	8061250.56	4483972.49	5999664.29	5541053.38
Q80T41	Gamma-aminobutyric acid type B receptor subunit 2	5986145.85	7889734.64	11334076.2	8833452.86	12843363.3	4830508.46	4669530.69	6729391.14	4258695.02	8761525.98	8298532.62	11520992.2	7893577.72	10858856.5
O88952	Protein lin-7 homolog C	7442220.7	5668922.39	6233255.85	6028368.87	5834705.69	4235140.34	4673537.94	4972431.54	4792841.55	5129618.33	4718354.1	4598330.02	6723029.03	5500402.51
Q9CYG7	Mitochondrial import receptor subunit TOM34	8448735.12	6349520.62	7621974.55	4759024.67	8475902.87	4145775.79	5255009.55	5027227	3811235.05	5126349.59	4586696	7136704.57	4549194.16	8302007.08
Q9QZ08	N-acetyl-D-glucosamine kinase	6342693.59	4582047.44	5328551.22	6485662.81	5063533.23	3827388.78	3663687.86	5189481.84	4493968.03	5287875.93	5313930.49	4456566.58	5541459.59	5802902.32
Q6PFQ7	Ras GTPase-activating protein 4	5693273.24	6634468.13	5013963.14	5038954.54	4664002.64	4553390.09	4302815.56	4050457.69	4072192.63	3256032.47	3322411.81	5601483.41	4663517.41	3580893.7
Q80TY0	Formin-binding protein 1	5507564.23	4260786.33	4003276.29	3971735.9	3942434.54	3538664.28	3367189.76	3232387.04	3866053.32	5028590.34	2485962.26	3195128.83	3405988.67	2482746.41
Q9QXT0	Protein canopy homolog 2	4554795.32	3748648.49	4826819.73	3849618.46	4741331.67	3650881.65	3401979.9	3640566.98	3057106.43	3223745.42	3283534.48	2574039.2	4127869.17	3982242.03

Accession Number	Protein Name	bgi wt 1	bgi wt 2	bgi wt 3	bgi wt 4	bgi wt 5	bgi $ERK2^{wt}$ 1	bgi $ERK2^{wt}$ 2	bgi $ERK2^{wt}$ 3	bgi $ERK2^{wt}$ 4	bgi $RKIP^{wt}$ 1	bgi $RKIP^{wt}$ 2	bgi $RKIP^{S153A}$ 1	bgi $RKIP^{S153A}$ 2	bgi $RKIP^{S153A}$ 3
Q9WVT6	Carbonic anhydrase 14	2247264.35	2504125.69	2612083.81	1230209.73	827753.077	4121632.56	3207475.86	2793090.08	3533567.79	4366748.99	992002.244	1655391.68	1657362.74	1689718.06
Q5BJ29	F-box/LRR-repeat protein 7	2998644.59	2638036.34	2482451.27	2030190.9	1690006.67	3489692.47	3706646.2	3507347.16	2504246.8	1617530.03	1165970.25	770589.903	1930865.9	1168793.12
Q91YP0	L-2-hydroxyglutarate dehydrogenase, mitochondrial	6416788.99	4965932.39	4802805.99	3238760.8	4610621.72	2555619.45	2913336.79	3581530.06	3981454.52	3710420.09	2578625.74	3793310.67	4522424.34	4157109.88
Q8R0F8	Acylpyruvase FAHD1, mitochondrial	6092908.98	3625314.95	5872903.8	3805034.39	7028888.79	2326811.48	3240928.53	3527271.56	3093556.9	6335343.11	5248984.96	4984899.3	5148224.08	5089011.83
Q9ERR1	Nuclear distribution protein nudE-like 1	3567721.76	4078234.86	2996042.43	4298074.75	4106920.19	2975630.44	2934923.03	2912488.17	3259203.44	2723630.92	3758041.37	3304160.78	3070572.12	2309473.84
O70311	Glycylpeptide N-tetradecanoyltransferase 2	738957.192	2938397.99	1949142.11	2177976.99	1602703.41	2720985.03	3248726.75	3272834.59	2370043.15	1400983.44	3101120.64	4004847.45	3362396.95	1374314.99
Q6NVG1	Lysophospholipid acyltransferase LPCAT4	647203.114	1389175.74	644962.909	1721416.13	964381.679	2465475.1	4124837.79	2182304.44	2412643.95	573940.936	3457346.06	2047855.61	1773994.87	915984.801
Q9CQJ6	Density-regulated protein	3969815.89	3627798.91	3197097.21	3568911.5	2875222.2	2748137.6	2468480.29	2814591.23	2953273.31	2748256.01	2721208.03	2799304.04	3183890.83	3245416.86
Q9CQ80	Vacuolar protein-sorting-associated protein 25	178943.768	1709473.35	1165867.57	1534182.84	1434159.09	3316499.2	3116419.21	2347500.57	1499869.56	1525046.52	2558190.26	2847281.26	1758934.04	1532663.42
P47964	60S ribosomal protein L36	3758467.19	2393241.08	4244014.84	3208263.83	4302919.48	2520721.97	1789337.51	2973613	2549543.09	3889369.9	2831098.87	4391969.93	3673685.8	2140524.39
Q8BH04	Phosphoenolpyruvate carboxykinase [GTP], mitochondrial	7210015.32	3071694.11	7211628.25	3133962.45	9927370.85	3006828.1	1301456.74	3171297.77	1915423	7654149.62	2447293.45	4601165.39	3253763.51	6205698.92
Q91WJ8	Far upstream element-binding protein 1	5413147.85	2932894.23	6678616.98	4243047.36	4687257.49	1651553.82	1707775.41	3273238.63	2346407.15	6802577.32	5605575.8	5733131.14	6666853.45	5037325.09
Q3UBX0	Transmembrane protein 109	2768489.49	3016845.6	3612087.16	2423568.13	2352027.53	1998484.14	2584506.12	2121672.67	1723575.56	1607682.31	1771701.92	2450574.29	2318235.17	1859646.37
P35288	Ras-related protein Rab-23	3480095.58	3469339.3	2763345.11	3468680.56	2492502.93	2383249.1	2125462.28	2189492.96	1508409.93	3364599.01	2866420.79	2836189.4	2334649.1	2671363.12

Accession Number	Protein Name	bgi wt 1	bgi wt 2	bgi wt 3	bgi wt 4	bgi wt 5	bgi ERK2^{wt} 1	bgi ERK2^{wt} 2	bgi ERK2^{wt} 3	bgi ERK2^{wt} 4	bgi RKIP^{wt} 1	bgi RKIP^{wt} 2	bgi RKIP^{S153A} 1	bgi RKIP^{S153A} 2	bgi RKIP^{S153A} 3
Q3KNM2	E3 ubiquitin-protein ligase MARCH5	3018549.99	2463101.71	2508864.25	2712469.01	2368801.22	1939793.31	1890108.81	1715658.09	2187515.22	2139499.27	2101885.76	1591755.64	1511324.39	1319327.07
P62838	Ubiquitin-conjugating enzyme E2 D2	1197803.25	1335044.58	1035270.76	1605490.86		1578210.81	1942357.5	2307503.13	1553100.4	1729179.07	2381165.89	1288705.96	1988736.15	1609721.84
Q8K1N1	Calcium-independent phospholipase A2-gamma	2950330.35	2328877.7	2671964.77	2604097.38	2364908.88	1748423.69	1712857.97	1782010.57	1538722.44	2180708.89	2274647.1	2040431.43	2544875.07	1899842.64
Q9D554	Splicing factor 3A subunit 3	1825199.06	2016206.39	3494172.85	2440933.63	3057638.81	1284171.85	1625985.44	1641229.11	2023951.04	3048037.61	2031146.43	2991911.91	2479092.84	2833982.41
P34152	Focal adhesion kinase 1		1023746.92	1463434.78	1008993.03	878644.697	1755701.6	1376646.21	1772398.73	1395976.68	977503.969	2803199.66	3383930.61	1645175.9	1858069.41
O35239	Tyrosine-protein phosphatase non-receptor type 9	3464269.24	2450740.63	2333892.57	2376514.54	1981670.57	1521676.54	1344159.72	1985127.7	1431001.92	1881802.44	1728349.38	1771914.09	2297440.74	2301286.79
Q8VBY2	Calcium/calmodulin-dependent protein kinase kinase 1	2288491.75	2257295.99	2724723.69	1970466.24	2588447.83	1368749.05	1532504.15	1305897.13	1880046.54	2074843.14	1979524.2	3470939.4	1253052.02	2687073.89
A6H5Z3	Exocyst complex component 6B	2098421.07	1885265.73	1953286.82	1673713.14	2031716.7	1479432.86	1244113.13	1536658.09	1450819.4	1675849.22	2024001.37	2818684.19	2026043.25	2152426.63
Q9CZR8	Elongation factor Ts, mitochondrial	2068467.89	1641471.9	1536702.65	1591136.01	1640335.25	1462543.18	1500240.12	1404316.66	1215120.15	1715824.13	1457193.68	1486473.99	1897380.93	1515716.68
Q9JHL1	Na(+)/H(+) exchange regulatory cofactor NHE-RF2	703553.382	538976.52	540784.364	548156.847	409580.346	1021105.95	1205771.96	1851700.01	1246630.26	535348.525	646615.983	1383726.11	1831861.77	1958345.69
Q9D0J4	ADP-ribosylation factor-like protein 2	1921749.8	1642144.41	1747736.91			1522156.95	1504695.55	1242742.95	1031177.77	1584812.07		1474190.07		1409546.47
P0DP60	Ly-6/neurotoxin-like protein 1	2087181.69	1419647.28	1825049.39	1609024.89	1429954.85	1202989.19	1194304.72	1284775.87	1470570.3	1880038.31	1479006.04	1930906.03	753661.921	1611635.66
O54724	Caveolae-associated protein 1	1704442.99	1932487.22	2859134.71	1512894.45	1812802.09	1144812.46	1240414.9	1252579.71	1369828.46	2990739.66	932292.089	1421699.07	3420714.48	1405737.7
Q8BFU3	RING finger protein 214	4122724.98	2459252.28	2248019.65	2029239.47	2300229.39	1225283.76	542696.695	2047570.51	1182102.34	1301296.63	1725681.61	2002761.78	1921516.21	358259.037
Q3TFD2	Lysophosphatidylcholine acyltransferase 1	679369.419	693467.502	537650.053	620259.029	1268530.69	1106933.72	1215475.87	1482821.56	929187.008	1347062.38	539276.173	862843.459	2922367.06	2178651.78

Accession Number	Protein Name	bgi wt 1	bgi wt 2	bgi wt 3	bgi wt 4	bgi wt 5	bgi ERK2^{wt} 1	bgi ERK2^{wt} 2	bgi ERK2^{wt} 3	bgi ERK2^{wt} 4	bgi RKIP^{wt} 1	bgi RKIP^{wt} 2	bgi RKIP^{S153A} 1	bgi RKIP^{S153A} 2	bgi RKIP^{S153A} 3
Q8CHX7	Raftlin-2	1473039.11	1839558.32	2130988.11	1647885.15	1591377.85	1020662.79	737604.709	1485934.34	1419932.85	1353419.44	969256.875	619124.87	1460010.46	2141063.49
Q6PDG5	SWI/SNF complex subunit SMARCC2	1468488.43	1699493.26	1670127	1252189.05	2310783.65	1102927.19	1291378.63	1093049.97	1093141.71	1427209.49	1614113.09	1734854.65	1645137.22	1963735.6
Q8BW41	Protein O-linked-mannose beta-1,4-N-acetylglucosaminyltransferase 2	2507872.49	1791659.52	1954857.11	1440748.24	1359662.75	1217724.4	952165.376	1588114.69	703437.847	1950304.37	1765806.09	886817.2	1515267.49	6061549.32
P11438	Lysosome-associated membrane glycoprotein 1	7877043.17	7263578.43	5934319.94	1189927.18	11695807.8	1099116.9	1118887.54	1046171.79	1058006.37	1057191.56	697628.356	695723.275	1016255.01	9275977.77
Q9QY23	Plakophilin-3	300339.749	712723.747	597034.875	893319.85	754108.825	984855.813	1100840.38	1073419.66	794977.367	601071.915	446629.261	615445.527	615275.839	788690.153
Q9CYK1	Tryptophan--tRNA ligase, mitochondrial	1234460.83	1137250.29	1012136.13	1133212.53	952856.868	856724.219		1007267.84	817311.42	946645.871	1459809.82	681172.219	885272.269	1060132.22
Q99M28	RNA-binding protein with serine-rich domain 1	909317.691	1373528.24	1613587.27	1281998.21	1157906.5	744618.144	763831.18	967231.514	1012524.6	1221059.35	1210198.49	1486371.77	1423182.11	1408988.32
Q9Z2Q5	39S ribosomal protein L40, mitochondrial	978270.54	1225441.2	943990.899	1116359.27	1012127.74	957554.095	747572.174	833827.313	911342.755	1215405.97	1087284.62	1070002.85	1398211.26	752967.975
Q920Q4	Vacuolar protein sorting-associated protein 16 homolog	1275779.4	1248729.37	1118457.39	1010265.62	823510.409	723769.733	880548.452	992953.463	775072.308	996948.331	1344602.55	1006166.1	887088.812	911012.522
Q8VD75	Huntingtin-interacting protein 1	660706.074	476890.69	587187.957	648020.988	503598.098	874101.944	670590.881	916095.941	662786.076	425460.61	870518.741	629796.349	936066.168	516817.083
Q8VD62	UPF0696 protein C11orf68 homolog	951575.653	1349059.23	1100334.5	1542057.36	1271459.26		766176.986	781714.56		1407745.26	1140202.46	1768369.09	1376344.05	1285331.17
B2RUJ5	Amyloid-beta A4 precursor protein-binding family A member 1	1863163.54	1658571.69	1108254.14	1979839.19	724868.617	511301.201	860507.399	813903.443	827251.705	1291350.53	1942129.67	1385325.06	1363953.26	1118230.25

Accession Number	Protein Name	bgi wt 1	bgi wt 2	bgi wt 3	bgi wt 4	bgi wt 5	bgi ERK2^{wt} 1	bgi ERK2^{wt} 2	bgi ERK2^{wt} 3	bgi ERK2^{wt} 4	bgi RKIP^{wt} 1	bgi RKIP^{wt} 2	bgi RKIP^{S153A} 1	bgi RKIP^{S153A} 2	bgi RKIP^{S153A} 3
Q9D1P0	39S ribosomal protein L13, mitochondrial	96692<br>1.639	18981<br>43.89	16414<br>30.39	12931<br>05.65	13047<br>28.82	52232<br>5.292	49135<br>5.572	91660<br>9.376	80868<br>9.757	60541<br>4.861	14155<br>30.43	54099<br>7.964	13293<br>32.25	13994<br>10.68
Q8R1S0	Ubiquinone biosynthesis monooxygenase COQ6, mitochondrial	11118<br>10.94	14667<br>33.14	15988<br>59.27	94921<br>2.183	24077<br>56.51	22863<br>8.092	51248<br>2.577	10051<br>37.31	96808<br>1.296	15558<br>08.02	11598<br>95.58	94637<br>8.483	80037<br>6.836	25932<br>63.87
Q9QUJ7	Long-chain-fatty-acid--CoA ligase 4	11051<br>42.29	85425<br>4.501	93519<br>5.515	71274<br>2.495	11335<br>52.88	49107<br>7.138	82707<br>1.778	67025<br>9.726	69019<br>9.521	78812<br>4.236	85784<br>4.806	11163<br>18.83	99398<br>8.596	12087<br>14.61
E9Q555	E3 ubiquitin-protein ligase RNF213	98350<br>3.747		72552<br>3.502	10566<br>77.68	10626<br>88.63	82541<br>1.298	59774<br>3.595	56710<br>9.45	59720<br>6.087	63657<br>3.078	74100<br>2.432	69590<br>4.328	72693<br>4.734	54787<br>7.685
Q80YA7	Dipeptidyl peptidase 8	10125<br>80.63	77586<br>2.365	89689<br>2.218	70694<br>5.9	92944<br>8.312	65004<br>0.915	54906<br>6.222	80333<br>9.437	58501<br>0.01	76537<br>0.543	62658<br>1.49	89152<br>4.518	77700<br>7.274	75499<br>4.969
P97372	Proteasome activator complex subunit 2	16605<br>54.65	97629<br>1.071	13498<br>09.04	10926<br>33.33	19157<br>86.31	45518<br>0.278	33387<br>5.629	12866<br>72.41	42723<br>9.841	13353<br>41.24			15316<br>07.18	13596<br>40.44
Q8K019	Bcl-2-associated transcription factor 1	48374<br>9.176		40861<br>3.019	34511<br>3.4				62763<br>9.647	61888<br>4.777	41489<br>4.983	52561<br>2.952	60921<br>9.825	56523<br>1.389	80581<br>9.452
O88845	A-kinase anchor protein 10, mitochondrial	69512<br>9.659	81858<br>9.588	69269<br>0.393	83559<br>9.543	65505<br>0.432	51020<br>3.422	55940<br>8.554	62028<br>5.642	57531<br>5.952	61891<br>6.418	72409<br>3.926	97392<br>7.376	58213<br>2.496	42835<br>5.935
Q9Z275	Retinaldehyde-binding protein 1	22589<br>1.154	16086<br>0.167	37140<br>4.762	26033<br>3.209	41577<br>1.413	64124<br>8.439	43216<br>7.758	73952<br>0.755	42843<br>0.469	26967<br>4.328	36726<br>6.169	67005<br>0.252	11077<br>76.92	37142<br>8.038
P59644	Phosphatidylinositol 4,5-bisphosphate 5-phosphatase A	87594<br>0.552	10021<br>12.28	51120<br>2.986	68960<br>1.631	10051<br>59.18	43894<br>5.767	60171<br>0.566	63615<br>7.662	50577<br>1.801	10990<br>29.75	10776<br>79.92	18091<br>03.85	12018<br>12.9	54666<br>7.028
P27671	Ras-specific guanine nucleotide-releasing factor 1	30817<br>09.55	17594<br>74.47	21740<br>60.31		24419<br>55.45	31661<br>7.624	29243<br>6.06	99138<br>0.844		16477<br>15.76	58614<br>7.679	27744<br>92.93	18102<br>69.04	20467<br>54.75
Q64455	Receptor-type tyrosine-protein phosphatase eta	81245<br>9.942	70919<br>3.34	57970<br>1.861	58516<br>0.427	70992<br>9.997	44684<br>1.801	53009<br>3.02	63025<br>9.346	51343<br>3.623	50297<br>6.956	63745<br>2.212	98643<br>1.567	68497<br>9.908	57107<br>3.241
Q3U2A8	Valine--tRNA ligase, mitochondrial		32270<br>1.303		37124<br>7.914			48990<br>1.186	54128<br>7.358	49327<br>7.723		56756<br>8.117	39990<br>3.356	36876<br>3.481	35588<br>2.321

Accession Number	Protein Name	bgi wt 1	bgi wt 2	bgi wt 3	bgi wt 4	bgi wt 5	bgi ERK2^{wt} 1	bgi ERK2^{wt} 2	bgi ERK2^{wt} 3	bgi ERK2^{wt} 4	bgi RKIP^{wt} 1	bgi RKIP^{wt} 2	bgi RKIP^{S153A} 1	bgi RKIP^{S153A} 2	bgi RKIP^{S153A} 3
Q9JJJ7	Protein-serine O-palmitoleoyltransferase porcupine	571615.701	735983.676	986115.041	635314.925	1020182.19	516038.066	461051.888	590141.799	464102.869	443958.216	655435.654	713161.431	637165.129	635479.891
Q61233	Plastin-2	656831.795	777878.816	822193.983	574384.295	674682.787	491802.041	644729.154	415792.467	476033.066	459678.444	435640.646	717625.843	578645.565	283001.177
Q8BHS6	Armadillo repeat-containing X-linked protein 3	799132.554	506229.779	588068.277	698036.318	702898.536	564094.555	510362.919	498552.252	433334.152	592475.295	562265.602	828907.265	509807.508	707819.575
Q80WQ2	Protein VAC14 homolog	477238.956	137622.936	319708.018	140481.703	281941.672	581680.472	434531.279	476129.606	429371.843	660205.415	462061.502	643613.836	797633.971	317455.208
P52189	Inward rectifier potassium channel 4		798229.52		897780.144		559092.388	414504.082	243658.553	653701.107		1067637.3	224744.758	426894.55	
O35855	Branched-chain-amino-acid aminotransferase, mitochondrial		697792.341	869842.596	477233.251	745625.668		402953.544		444102.159	790819.678	855766.6		345285.442	
Q6AXF6	SID1 transmembrane family member 1		684527.013	725244.269	657519.796	1046604.75	253399.613	447766.725	482659.072	504663.647	695557.7	573849.618	584464.543	473191.355	1373162.54
Q8BPU7	Engulfment and cell motility protein 1	189351.106	178781.896	211471.826	171659.751	156848.783	416108.285	381306.211	395434.667	412041.794	350634.261	137179.593			88146.8417
Q9EPR4	Solute carrier family 23 member 2	704008.073	529578.491	941504.082	476887.234	682373.06	331339.575	271676.608	599035.945	377736.329	884385.439	543417.727	1045307.53	860529.836	822055.549
Q9Z0H3	SWI/SNF-related matrix-associated actin-dependent regulator of chromatin subfamily B member 1	398613.986	595579.075	637492.603	561196.951	354218.03	288629.07	355561.354	318579.642	430858.22			628836.464	517819.673	513786.16
Q9QZH6	Evolutionarily conserved signaling intermediate in Toll pathway, mitochondrial	493393.298	479289.527	363748.791	507717.614	367123.131	331266.272	315374.582	389671.23	298255.132	322348.042	355761.373	285725.556	410143.953	355611.458
Q8VEA4	Mitochondrial intermembrane	1494741.13	1027600.79		1152216.55	1501366.55	366314.113	286054.437				514472.419	1187598.67	1138435.54	1061763.09

Accession Number	Protein Name	bgi wt 1	bgi wt 2	bgi wt 3	bgi wt 4	bgi wt 5	bgi ERK2^{wt} 1	bgi ERK2^{wt} 2	bgi ERK2^{wt} 3	bgi ERK2^{wt} 4	bgi RKIP^{wt} 1	bgi RKIP^{wt} 2	bgi RKIP^{S153A} 1	bgi RKIP^{S153A} 2	bgi RKIP^{S153A} 3
	space import and assembly protein 40														
Q9DA80	Radial spoke head protein 3 homolog B	524387.234	303484.105	506096.556	364263.126	510220.252	314337.147	265208.056	283087.129	386441.221			831844.658	364688.577	476039.328
G5E8F4	Fucose-1-phosphate guanylyltransferase	233404.568	202553.194	170846.953	260720.956		275233.982	332831.628	261192.873	300445.019	193132.34	268767.163	342185.449	259146.468	
Q9WTS5	Teneurin-2	716605.632	758491.285	1037086.61		1085798.25	359416.617	438015.542	89681.1564	226123.359	496404.1	117322.635	288209.322	110793.447	506462.946
Q68FH4	N-acetylgalactosamine kinase	442429.083	367312.122	364102.373	247423.866	368589.92	223857.619	172920.292	313426.55	271885.444	202601.526	308944.959	360591.599	291848.843	369378.346
Q80UM7	Mannosyl-oligosaccharide glucosidase	1383880.3	1643315.94	3150606.83	614751.86	985120.508	224710.72	182417.006	329427.411	197305.446	592400.346	653903.875	894736.063	800086.68	575403.567
Q6DFW4	Nucleolar protein 58	686302.294	421502.799	663621.923	295655.873	399341.464	221150.52	240161.522		231328.349	577850.751	433390.858	326736.77	401111.059	614892.338
P01843	Ig lambda-1 chain C region	331022.115	541702.101	451782.327	486653.035	310102.465		98355.3361	78317.4968	296745.983	281040.892		888539.484	355387.858	285616.038
Q8C7D2	Protein cereblon	205437.746	154019.875	187273.964		171782.16		74047.0145	105504.353	137825.525		276190.46	263227.548	189153.695	164121.501
O35684	Neuroserpin	221466.044		168034.533		246227.767		65396.7977	100797.591	138888.426	126514.863	201953.276	378105.778	151375.64	139746.637

Table S5

Significantly regulated proteins in wt bgi vs. ERK2^{wt} bgi.

Mass spectrometric analyses of ipsilateral basal ganglia (bgi) of wild-type (wt; n = 5) mice and mice ubiquitously overexpressing ERK2^{wt} (ERK2^{wt}; n = 4), RKIP^{wt} (RKIP^{wt}; n = 2) or a phosphorylation-deficient mutant of RKIP^{S153A} (RKIP^{S153A}; n = 3) 24 h after transient middle cerebral artery occlusion. The table shows the individual expression values of the proteins that are significantly regulated between wt bgi and ERK2^{wt} bgi and

indicates the protein accession number and protein name; n-numbers represent samples of individual mice. For statistical analysis unpaired Students t-test was applied.

Table S6

Accession Number	Protein Name	ratio wt bgi vs. ERK2^{wt} bgi	p value
Q80UM7	Mannosyl-oligosaccharide glucosidase	6.66281506	0.03829903
P27671	Ras-specific guanine nucleotide-releasing factor 1	4.43185879	0.00380249
Q8VEA4	Mitochondrial intermembrane space import and assembly protein 40	3.96702525	0.00248794
Q8BH04	Phosphoenolpyruvate carboxykinase [GTP], mitochondrial	2.6017799	0.04433272
Q8R1S0	Ubiquinone biosynthesis monooxygenase COQ6, mitochondrial	2.22061321	0.03482747
Q6DFW4	Nucleolar protein 58	2.13654103	0.02713226
Q91WJ8	Far upstream element-binding protein 1	2.13431612	0.01165191
Q8BFU3	RING finger protein 214	2.10650319	0.02553551
O35684	Neuroserpin	2.08378943	0.02481991
Q9D1P0	39S ribosomal protein L13, mitochondrial	2.07502951	0.00716978
B2RUJ5	Amyloid-beta A4 precursor protein-binding family A member 1	1.9475036	0.03737297
Q6AXF6	SID1 transmembrane family member 1	1.84419071	0.0203785
Q80T41	Gamma-aminobutyric acid type B receptor subunit 2	1.83078821	0.02167961
Q8R0F8	Acylpyruvase FAHD1, mitochondrial	1.73441539	0.02553436
Q9EPR4	Solute carrier family 23 member 2	1.68850503	0.04016281
Q8BW41	Protein O-linked-mannose beta-1,4-N-acetylglucosaminyltransferase 2	1.62365432	0.04190221
Q8VD62	UPF0696 protein C11orf68 homolog	1.60592285	0.00977139
P57776	Elongation factor 1-delta	1.60358283	0.02566496
O54724	Caveolae-associated protein 1	1.56908567	0.03687263
Q9D554	Splicing factor 3A subunit 3	1.56148954	0.04020529
Q9JJJ7	Protein-serine O-palmitoleoyltransferase porcupine	1.55531698	0.03353774
Q8VBY2	Calcium/calmodulin-dependent protein kinase kinase 1	1.55466311	0.00266835
Q9Z268	RasGAP-activating-like protein 1	1.54504727	0.03845168
P35288	Ras-related protein Rab-23	1.52793471	0.00656646
Q60634	Flotillin-2	1.5265407	0.01550524
P59644	Phosphatidylinositol 4,5-bisphosphate 5-phosphatase A	1.4969461	0.04529764
Q8CHX7	Raftlin-2 OS=Mus musculus	1.48929636	0.03902729
Q91YP0	L-2-hydroxyglutarate dehydrogenase, mitochondrial	1.47544623	0.03875272
Q6PDG5	SWI/SNF complex subunit SMARCC2	1.46727842	0.03671458
Q68FH4	N-acetylgalactosamine kinase	1.45799879	0.03688863
P47964	60S ribosomal protein L36	1.45685051	0.03720649
Q99M28	RNA-binding protein with serine-rich domain 1	1.45320292	0.02532537
Q8R5M8	Cell adhesion molecule 1	1.44163242	0.02729474
Q8BMJ2	Leucine--tRNA ligase, cytoplasmic	1.43814927	0.02327837

Accession Number	Protein Name	ratio wt bgi vs. ERK2^{wt} bgi	p value
Q9QUJ7	Long-chain-fatty-acid--CoA ligase 4	1.4159257	0.03227205
O08917	Flotillin-1	1.40795537	0.01721571
Q99JY8	Phospholipid phosphatase 3	1.39440569	0.04264189
P63044	Vesicle-associated membrane protein 2	1.38098511	0.04248087
P70206	Plexin-A1	1.37581235	0.02286428
Q9ESJ4	NCK-interacting protein with SH3 domain	1.36822401	0.03030421
Q3UUG6	TBC1 domain family member 24	1.36422943	0.00860174
Q8C0L0	Thioredoxin-related transmembrane protein 4	1.361776	0.03265431
Q9DCX2	ATP synthase subunit d, mitochondrial	1.36043051	0.03063373
A6H5Z3	Exocyst complex component 6B	1.35070759	0.00130775
Q80YA7	Dipeptidyl peptidase 8	1.3362093	0.02838719
Q64332	Synapsin-2	1.33500696	0.02432841
Q8BHS6	Armadillo repeat-containing X-linked protein 3	1.31357959	0.03409097
O88845	A-kinase anchor protein 10, mitochondrial	1.30568161	0.00607993
P0DP60	Ly-6/neurotoxin-like protein 1	1.29966122	0.03607945
Q920Q4	Vacuolar protein sorting-associated protein 16 homolog	1.29921319	0.04401468
Q9QZ08	N-acetyl-D-glucosamine kinase	1.29505699	0.04150132
Q9QXZ0	Microtubule-actin cross-linking factor 1	1.28720518	0.04607829
Q64455	Receptor-type tyrosine-protein phosphatase eta	1.28129815	0.03673653
Q9CPU0	Lactoylglutathione lyase	1.28006933	0.04308695
Q99JR1	Sideroflexin-1	1.2732441	0.03276428
Q9ERR1	Nuclear distribution protein nudE-like 1	1.26115594	0.02595867
P60764	Ras-related C3 botulinum toxin substrate 3	1.25356492	0.02598038
Q8BLK3	Limbic system-associated membrane protein	1.25170961	0.01641169
Q9ER00	Syntaxin-12	1.23703609	0.04110565
O88545	COP9 signalosome complex subunit 6	1.22448628	0.02705103
Q9CYK1	Tryptophan--tRNA ligase, mitochondrial	1.22401288	0.04938957
Q9Z2Q5	39S ribosomal protein L40, mitochondrial	1.22335919	0.02688828
Q9CPQ8	ATP synthase subunit g, mitochondrial	1.22057497	0.02401955
Q9CRD0	OCIA domain-containing protein 1	1.22026939	0.01605864
Q8BMF4	Dihydrolipoyllysine-residue acetyltransferase component of pyruvate dehydrogenase complex, mitochondrial	1.21980135	0.02983246
Q9CZR8	Elongation factor Ts, mitochondrial	1.21501675	0.03601036
Q62418	Drebrin-like protein	1.20803357	0.04768027
P34022	Ran-specific GTPase-activating protein	1.17699174	0.04236046
Q80U28	MAP kinase-activating death domain protein	1.1728895	0.02479982
Q9Z0Y1	Dynactin subunit 3	1.17159959	0.02907167
Q3V3R1	Monofunctional C1-tetrahydrofolate synthase, mitochondrial	1.16796713	0.01763236
Q9CQA3	Succinate dehydrogenase [ubiquinone] iron-sulfur subunit, mitochondrial	1.15449387	0.04817165
P16332	Methylmalonyl-CoA mutase, mitochondrial	1.15138734	0.00617996

Accession Number	Protein Name	ratio wt bgi vs. ERK2^{wt} bgi	p value
Q9D051	Pyruvate dehydrogenase E1 component subunit beta, mitochondrial	1.14601791	0.01026793
P17710	Hexokinase-1	1.14375239	0.02904563
P62814	V-type proton ATPase subunit B, brain isoform	1.14103166	0.02442357
Q9D7N9	Adipocyte plasma membrane-associated protein	1.13853345	0.03511707
P14094	Sodium/potassium-transporting ATPase subunit beta-1	1.13717646	0.0456891
Q9D6J6	NADH dehydrogenase [ubiquinone] flavoprotein 2, mitochondrial	1.1260775	0.02360965
Q8QZT1	Acetyl-CoA acetyltransferase, mitochondrial	1.12600869	0.0466675
Q9D8B4	NADH dehydrogenase [ubiquinone] 1 alpha subcomplex subunit 11	1.11712064	0.03866276
Q91VD9	NADH-ubiquinone oxidoreductase 75 kDa subunit, mitochondrial	1.10587947	0.04836217
Q99PL6	UBX domain-containing protein 6	0.85978686	0.04768428
G5E8K5	Ankyrin-3	0.82449484	0.03287138
P12787	Cytochrome c oxidase subunit 5A, mitochondrial	0.81721238	0.03297267
P68369	Tubulin alpha-1A chain	0.81426521	0.03057077
Q9EPW0	Inositol polyphosphate-4-phosphatase type I A	0.79912343	0.02114599
Q99LB6	Methionine adenosyltransferase 2 subunit beta	0.76158698	0.03553057
Q9R1V7	Disintegrin and metalloproteinase domain-containing protein 23	0.74421413	0.04374519
G5E8F4	Fucose-1-phosphate guanylyltransferase	0.74166288	0.0245185
Q8VD75	Huntingtin-interacting protein 1	0.73669534	0.04456282
Q04447	Creatine kinase B-type	0.72307417	0.03987471
Q5BJ29	F-box/LRR-repeat protein 7	0.71710419	0.03671851
P62838	Ubiquitin-conjugating enzyme E2 D2	0.7009198	0.04773969
P34152	Focal adhesion kinase 1	0.69433607	0.0292329
P28661	Septin-4	0.68656624	0.0215601
Q3U2A8	Valine--tRNA ligase, mitochondrial	0.68281198	0.03384065
Q80YN3	Breast carcinoma-amplified sequence 1 homolog	0.66432849	0.03742776
Q8K019	Bcl-2-associated transcription factor 1	0.66182717	0.03299244
Q9QY23	Plakophilin-3	0.65906935	0.02864339
Q8R016	Bleomycin hydrolase	0.65063061	0.01412568
O70311	Glycylpeptide N-tetradecanoyltransferase 2	0.64806753	0.04927239
O70172	Phosphatidylinositol 5-phosphate 4-kinase type-2 alpha	0.6462849	0.0055344
Q3TFD2	Lysophosphatidylcholine acyltransferase 1	0.64198414	0.04530113
P16330	2',3'-cyclic-nucleotide 3'-phosphodiesterase	0.63454531	0.039659
P19246	Neurofilament heavy polypeptide	0.62973457	0.04226027
Q8BZA9	Fructose-2,6-bisphosphatase TIGAR	0.62969275	0.01980239
P46660	Alpha-internexin	0.62018242	0.03313355
P08551	Neurofilament light polypeptide	0.60638433	0.03335447

Accession Number	Protein Name	ratio wt bgi vs. ERK2^{wt} bgi	p value
Q9D2P8	Myelin-associated oligodendrocyte basic protein	0.59124811	0.04068454
P08553	Neurofilament medium polypeptide	0.58182505	0.01828076
Q8C078	Calcium/calmodulin-dependent protein kinase kinase 2	0.56747845	0.04590781
Q80WQ2	Protein VAC14 homolog	0.5649098	0.02728618
Q9WVT6	Carbonic anhydrase 14	0.55193895	0.01238698
P56375	Acylphosphatase-2	0.54489107	0.01312531
Q9Z275	Retinaldehyde-binding protein 1	0.51192346	0.02908361
Q9CQ80	Vacuolar protein-sorting-associated protein 25	0.46867374	0.03666826
Q6NVG1	Lysophospholipid acyltransferase LPCAT4	0.38387227	0.02221692

Table S6

Exclusively significantly regulated proteins in wt bgi vs. ERK2^{wt} bgi.

Mass spectrometric analyses of ipsilateral basal ganglia (bgi) of wild-type (wt; n = 5) mice and mice ubiquitously overexpressing ERK2^{wt} (ERK2^{wt}; n = 4), RKIP^{wt} (RKIP^{wt}; n = 2) or a phosphorylation-deficient mutant of RKIP^{S153A} (RKIP^{S153A}; n = 3) 24 h after transient middle cerebral artery occlusion. The table shows the proteins that are significantly regulated in wt bgi vs. ERK2^{wt} bgi and not in RKIP^{wt} or RKIP^{S153A} vs. wt and indicates the protein accession number, protein name, ratio and p-values; n-numbers represent samples of individual mice. For statistical analysis unpaired Students t-test was applied.

Table S7

Accession Number	Protein Name	Ratio wt bgi vs. RKIP^{wt} bgi	p value of wt bgi vs. RKIP^{wt} bgi	Ratio wt bgi vs. RKIP^{S153A} bgi	p value of wt bgi vs. RKIP^{S153A} bgi
Q9JIA9	Cathepsin R	2.18181359	0.03552668	2.07855741	0.02854479
O08848	60 kDa SS-A/Ro ribonucleoprotein	2.06608535	0.02048749	1.75562149	0.03764666
Q9D8S4	Oligoribonuclease, mitochondrial	1.82950355	0.00448633	3.47302242	0.0009357
Q3TBW2	39S ribosomal protein L10, mitochondrial	1.69246847	0.02604891	1.80664302	0.01959881
Q3UJU9	Regulator of microtubule dynamics protein 3	1.66239854	0.00666483	1.36712463	0.04933758
Q9R1Q9	V-type proton ATPase subunit S1	1.65493032	0.03037567	1.3223843	0.01809522
Q9WUL7	ADP-ribosylation factor-like protein 3	1.55114397	0.04246911	1.54477833	0.04285244
O35864	COP9 signalosome complex subunit 5	1.32204382	0.02774688	1.33930991	0.01777945
Q9CZ42	ATP-dependent (S)-NAD(P)H-hydrate dehydratase	1.24848703	0.00289857	1.11426516	0.03290119
P47791	Glutathione reductase, mitochondrial	1.22539659	0.02946901	1.20308208	0.03956764
P32883	GTPase KRas	0.83617566	0.03185616	0.86447375	0.03412525
Q8BMJ3	Eukaryotic translation initiation factor 1A, X-chromosomal	0.7508331	0.00915363	0.80348236	0.03958311
Q9DCB1	High mobility group nucleosome-binding domain-containing protein 3	0.45775327	0.01068347	0.63806344	0.02051965

Table S7

Exclusively significantly regulated proteins in wt bgi vs. RKIP^{wt} bgi and wt bgi vs. RKIP^{S153A}.

Mass spectrometric analyses of ipsilateral basal ganglia (bgi) of wild-type (wt; n = 5) mice and mice ubiquitously overexpressing ERK2^{wt} (ERK2^{wt}; n = 4), RKIP^{wt} (RKIP^{wt}; n = 2) or a phosphorylation-deficient mutant of RKIP^{S153A} (RKIP^{S153A}; n = 3) 24 h after transient middle cerebral artery occlusion. The table shows the proteins that are significantly regulated in wt bgi vs. RKIP^{wt} and RKIP^{S153A} and not in ERK2^{wt} vs. wt and indicates the protein accession number,

protein name, ratio and p-values; n-numbers represent samples of individual mice. For statistical analysis unpaired Students t-test was applied.

Table S8

wt bgi vs. ERK2^wt bgi	
up-regulated terms in wt	***up-regulated terms in ERK2^{wt}***
rRNA modification	Phagocytosis
snRNP protein import into nucleus	Engulfment
Ribosome biogenesis	Apoptotic process
snoRNA biogenesis	Movement of cell or subcellular component
Small GTPase mediated signal transduction	Cell migration
Cell proliferation	Rac protein signal transduction
Neuron projection development	Actin filament-based process
Regulation of Rac, Ras and Rho protein signal transduction	Actin cytoskeleton organization
Intracellular signal transduction	(Phospho)lipid metabolic process
Positive regulation of GTPase activity	Phospholipid biosynthetic process
Positive regulation of Ras protein signal transduction	Sensory perception of sound
Positive regulation of fibroblast proliferation	Negative regulation of phosphatidylinositol 3-kinase signalling
Regulation of (neuronal) synaptic plasticity	Positive regulation of membrane potential
Regulation of N-methyl-D-aspartate selective glutamate receptor activity	Cell-cell adhesion
Ubiquinone biosynthetic process	Transcription, DNA-templated
Secondary metabolite biosynthetic process	(Protein) transport
Oxidation-reduction process	Protein targeting to vacuole involved in ubiquitin-dependent protein catabolic process via the multivesicular body sorting pathway
(Protein) transport	
Protein maturation by protein folding	
Cellular response to oxidative stress	
Mitochondrial DNA repair	
Protein import into mitochondrial intermembrane space	
Regulation of protein export from nucleus	
‘de novo’ posttranslational protein folding	
Establishment of protein localization to mitochondrion/organelle	
Positive regulation of cellular respiration	
(Regulation of) transcription, DNA-templated	
(Negative) regulation of transcription from RNA polymerase II promotor	
Positive regulation of gene expression	
Cell growth	
Stem cell division	
Hematopoietic stem cell proliferation	
Autophagy	
Spermatogenesis	
Granzyme-mediated apoptotic signaling pathway	
Positive regulation of natural killer cell degranulation/mediated cytotoxicity	
Autophagic cell death	

Protein stabilization	
Golgi to lysosome transport	
Regulation of organelle transport along microtubule	
Protein N-linked glycosylation	
(Oligocaccharide) metabolic process	
Translation	
Pyruvate/Oxaloacetate metabolic process	
Gluconeogenesis	
NADH oxidation	
Positive regulation of insulin secretion	
Response to lipopolysaccharide	
Cellular response to glucose stimulus/TNF	
Response to dexamethasone	
Antigen processing and presentation of exogenous antigen	
Regulation of proteasomal protein catabolic process	
Regulation of G1/S transition of mitotic cell cycle	
Negative regulation of peptidase activity	
Regulation of cell adhesion	
Cell morphogenesis	
(Heterophilic) cell adhesion (via plasma membrane cell adhesion molecules)	
Signal transduction	
Axon guidance	
Single organismal cell-cell adhesion	
Calcium-mediated signalling using intracellular calcium source	
Neuron development	
Positive regulation of filopodium assembly	
Self proteolysis	
**wt bgi vs. RKIP^{wt} bgi**	
up-regulated terms in wt	***up-regulated terms in RKIP^{wt}***
Localization	(m/t)RNA processing
(Regulation of) transcription, DNA-templated	Anterior/posterior pattern specification
(Negative/positive) regulation of transcription from RNA polymerase II promoter	Morphogenesis of embryonic epithelium
(Protein) transport	Ovarian follicle development
Positive regulation of gene expression	Transcription elongation from RNA polymerase II promoter
Regulation of multivesicular body size involved in endosome transport	Ubiquitin-dependent protein catabolic process
Endocytic recycling	Spermatid development
(Positive) regulation of protein catabolic process	Regulation of T cell differentiation in thymus
Protein targeting to vacuole involved in ubiquitin-dependent protein catabolic process via the multivesicular body sorting pathway	Follicle-stimulating hormone signaling pathway
Early endosome to late endosome transport	Proteasome assembly
Regulation of protein complex stability	Oogenesis
Positive regulation of exosomal secretion	Thymus development

Regulation of viral budding via host ESCRT complex	Sertoli cell development
Immune system development	Positive regulation of growth hormone receptor signaling pathway
Smoothened signaling pathway	Adipose tissue development
Response to UV	Seminiferous tubule development
Cell projection organization	Neuron migration
Cilium morphogenesis	tRNA wobble uridine modification
Kidney development	(Regulation of) transcription, DNA-templated
Renal water homeostasis	(Positive) regulation of transcription from RNA polymerase II promoter
Visual perception	(Central) nervous system development
Sensory perception of sound	Positive regulation of cell migration
ER-associated ubiquitin-dependent protein catabolic process	Histone H3/H4 acetylation
ER calcium ion homeostasis	Liver development
Response to ER stress	Immune system process
Olfactory behavior	Proteolysis
Glucose homeostasis	Acute-phase response
Negative regulation of programmed cell death	Notch signalling pathway
Negative regulation of sequence-specific DNA binding transcription factor activity	Positive regulation of cell death
Negative regulation of neuron apoptotic process	Response to hydrogen peroxide
Positive regulation of adenylate cyclase activity	Defense response to bacterium
Positive regulation of cell growth	Negative regulation of oxidoreductase activity
Protein stabilization	Negative regulation of hydrogen peroxide catabolic process
Neurological system process	Covalent chromatin modification
Positive regulation of protein metabolic process	Positive regulation of sequence-specific DNA binding transcription factor activity
(Regulation of) cell cycle	Regulation of insulin secretion involved in cellular response to glucose stimulus
Positive regulation of calcium ion transport	(intracellular protein) transport
Calcium ion homeostasis	Protein import into mitochondrial matrix
Negative regulation of ER stress-induced intrinsic apoptotic signaling pathway	Protein import into mitochondrial outer membrane
Negative regulation of ATF6-mediated unfolded protein response	
Negative regulation of type B pancreatic cell apoptotic process	
Long-chain fatty acid metabolic process	
Acyl-CoA metabolic process	
Proteolysis	
Protein catabolic process	
Proteolysis involved in cellular protein catabolic process	
(Positive) regulation of cyclin-dependent protein serine/threonine kinase activity	
G2/M transition of mitotic cell cycle	
(Regulation of canonical) Wnt signaling pathway	

Cell division	
Mitochondrial electron transport	
Cytochrome c to oxygen	
FAD biosynthetic process	
Metabolic process	
(Cortical actin) cytoskeleton organization	
Cellular component organization	
Regulation of cell morphogenesis	
Cell-cell adhesion	
Protein import into nucleus	
NLS-bearing protein import into nucleus	
(Protein) transport	
Maternal process involved in female pregnancy	
Autophagy	
Spermatogenesis	
Granzyme-mediated apoptotic signalling pathway	
Positive regulation of natural killer cell degranulation/mediated cytotoxicity	
Autophagic cell death	
Protein stabilization	
Establishment of protein localization to organelle	
Golgi to lysosome transport	
Regulation of organelle transport along microtubule	
**wt bgi vs. RKIP^{S153A} bgi**	
up-regulated terms in wt	***up-regulated terms in RKIP^{S153A}***
Fatty acid biosynthetic/metabolic process	Epithelial cell development
Fatty acid beta-oxidation	Cilium movement
(Transmembrane) transport	Multicellular organism development
Peroxisome organization	Brain development
Response to organic cyclic compound	Ventricular system development
Peroxisomal long-chain fatty acid import	Trachea development
Response to drug	Axonemal central apparatus assembly
Very long-chain fatty acid catabolic process	(Protein) transport
Cell activation	Oxidation-reduction process
Negative regulation of activation of membrane attack complex	Oocyte maturation
Negative regulation of angiogenesis	Endosomal transport
Negative regulation of vascular endothelial growth factor receptor signaling pathway	Polar body extrusion after meiotic divisions
Negative regulation of complement activation	Spindle assembly involved in meiosis
Negative regulation of fibroblast growth factor production	Heme catabolic process
Nucleobase-containing compound metabolic process	(Regulation of) transcription, DNA-templated
Nucleic acid phosphodiester bond hydrolysis	mRNA export from nucleus
Proteolysis (involved in cellular protein catabolic process)	Positive regulation of cell proliferation
Protein catabolic process	Positive regulation of histone methylation
Cell differentiation	Positive regulation of ATPase activity
Positive regulation of dendrite extension	Positive regulation of helicase activity
GPCR signaling pathway	Protein deubiquitination

Purine ribonucleoside diphosphate catabolic process	(Protein) phosphorylation
Platelet activation	Transmembrane receptor protein tyrosine kinase signalling pathway
Protein homooligomerization	Cell migration
Cellular response to IL-6	Cell differentiation
DNA (unwinding involved in) replication (initiation)	Peptidyl-tyrosine autophosphorylation
Cell cycle	Regulation of cell proliferation
Skeletal muscle contraction	Innate immune response
Skeletal muscle tissue development	(Cellular) response to hypoxia/ischemia
Sensory perception of sound	Blood vessel remodeling
Regulation of cell shape	mRNA splice site selection
Neuronal action potential	mRNA processing
Actin filament-based movement	Regulation of gene expression
Actomyosin structure organization	Cardiac muscle cell apoptotic process
Mitochondrion morphogenesis	Negative regulation of striated muscle cell apoptotic process
Vocalization behavior	Negative regulation of TNF-mediated signaling pathway
Glucose metabolic process	(Regulation of) release of sequestered calcium ion into cytosol by SR
(Regulation of) transcription, DNA-templated	Negative regulation of muscle atrophy
Protein phosphorylation	Response to injury involved in regulation of muscle adaptation
Lipid metabolic/biosynthetic process	Regulation of NF-kappaB import into nucleus
Fatty acid metabolic process	(Negative) regulation of apoptotic process
Steroid/cholesterol/sterol biosynthetic process	Negative regulation of cysteine-type endopeptidase activity (involved in apoptotic process)
(Positive regulation of) autophagy	Myoblast differentiation
Steroid/cholesterol metabolic process	Smooth muscle cell proliferation
Positive regulation of cell proliferation	Protein oligomerization
Response to UV/gamma radiation	Negative regulation of cellular/mitochondrial calcium ion concentration
Cold acclimation	Negative regulation of necrotic cell death
Positive regulation of gene expression	Negative regulation of release of cytochrome c from mitochondria
Response to activity	Intrinsic apoptotic signaling pathway
Wnt signaling pathway	Negative regulation of extrinsic apoptotic signaling pathway (via death domain receptors)
Phosphorylation	Negative regulation of mitochondrial membrane permeability involved in apoptotic process
Covalent chromatin modification	Negative regulation of oxidative stress-induced intrinsic apoptotic signaling pathway
Fatty acid oxidation	Negative regulation of death-inducing signaling complex assembly

Response to caffeine	Negative regulation of hydrogen peroxide-induced cell death
Cellular response to nutrient levels/glucose starvation	Negative regulation of protein targeting to mitochondrion
Negative regulation of TOR signaling	Negative regulation of (hypoxia-induced) intrinsic apoptotic signaling pathway
Regulation of peptidyl-serine phosphorylation	Sensory perception of sound
(Cellular) response to hydrogen peroxide	Negative regulation of phosphatidyl 3-kinase signaling
Glucose/fatty acid homeostasis	Positive regulation of membrane potential
Regulation of circadian rhythm	Cell-cell adhesion
Negative regulation of apoptotic process	
Positive regulation of catabolic process	
Protein heterooligomerization	
Regulation of vesicle-mediated transport	
Cellular response to ethanol	
Cellular response to prostaglandin E stimulus	
Cellular response to organonitrogen compound	
Cellular response to hypoxia	
Response to camptothecin	
Regulation of energy homeostasis	
Negative regulation of glucose import in response to insulin stimulus	
G-protein coupled acetylcholine receptor signaling pathway	
Negative regulation of signal transduction	
Positive regulation of GTPase activity	
Regulation of dopamine receptor signaling pathway	
Vesicle fusion	
Acrosome reaction	
Regulation of calcium ion-dependent exocytosis	
Calcium ion-regulated exocytosis of neurotransmitter	
Acrosomal vesicle exocytosis	
Negative regulation of transcription from RNA polymerase II promoter	
Cell morphogenesis	
(Heterophilic cell-) cell adhesion (via plasma membrane cell adhesion molecules)	
Signal transduction	
Axon guidance	
Single organismal cell-cell adhesion	
Calcium-mediated signaling using intracellular calcium source	
Neuron development	
Positive regulation of filopodium assembly	
Self proteolysis	

Table S8

Biological processes regulated in wt bgi vs. ERK2^{wt} bgi, wt bgi vs. RKIP^{wt} bgi and wt bgi vs. RKIP^{S153A} bgi.

Expression levels of proteins from brains after 45 minutes of tMCAO were compared between the ipsilateral hemisphere of basal ganglia (bgi) from wt mice (n = 5) and mice with ubiquitous overexpression of ERK2^{wt} (ERK2^{wt}; n = 4), RKIP^{wt} (RKIP^{wt}; n = 2) or a phosphorylation-deficient mutant of RKIP^{S153A} (RKIP^{S153A}; n = 3). Mass spectrometry data with a cut-off of > 2 and < 0.05 were annotated to the Gene Ontology Term: Biological Process using the data from Uniprot.

Full unedited gels for Figure S1:

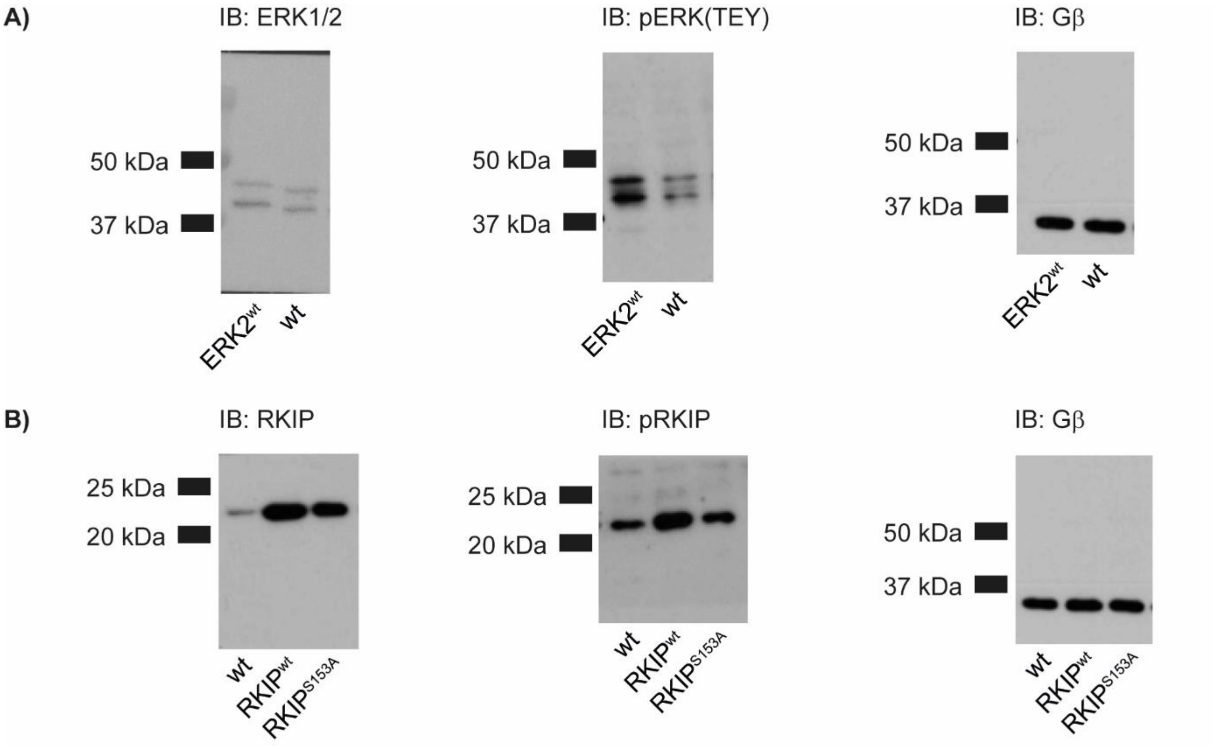

Supplement: Supplementary file 1 [file ijms-23-00706-s001.zip › ijms-1527488-supplementary.pdf]
